# Cure and death play a role in understanding dynamics for COVID-19: Data-driven competing risk compartmental models, with and without vaccination

**DOI:** 10.1371/journal.pone.0254397

**Published:** 2021-07-15

**Authors:** Min Lu, Hemant Ishwaran

**Affiliations:** Department of Public Health Sciences, Miller School of Medicine, University of Miami, Miami, FL, United States of America; Centers for Disease Control and Prevention, UNITED STATES

## Abstract

Several factors have played a strong role in influencing the dynamics of COVID-19 in the U.S. One being the economy, where a tug of war has existed between lockdown measures to control disease versus loosening of restrictions to address economic hardship. A more recent effect has been availability of vaccines and the mass vaccination efforts of 2021. In order to address the challenges in analyzing this complex process, we developed a competing risk compartmental model framework with and without vaccination compartment. This framework separates instantaneous risk of removal for an infectious case into competing risks of cure and death, and when vaccinations are present, the vaccinated individual can also achieve immunity before infection. Computations are performed using a simple discrete time algorithm that utilizes a data driven contact rate. Using population level pre-vaccination data, we are able to identify and characterize three wave patterns in the U.S. Estimated mortality rates for second and third waves are 1.7%, which is a notable decrease from 8.5% of a first wave observed at onset of disease. This analysis reveals the importance cure time has on infectious duration and disease transmission. Using vaccination data from 2021, we find a fourth wave, however the effect of this wave is suppressed due to vaccine effectiveness. Parameters playing a crucial role in this modeling were a lower cure time and a signficantly lower mortality rate for the vaccinated.

## Introduction

The coronavirus disease (COVID-19) first identified in Wuhan, China in late 2019 [1] rapidly spread across mainland China, and then across the globe, eventually manifesting itself into the global pandemic of 2020. Evidence from regions affected early in the pandemic suggested a high fatality rate, which combined with the highly infectious nature of COVID-19, spurred both national and international governments to impose strict measures to reduce its spread. These early attempts to contain the outbreak were partially effective. For example, lockdown measures implemented in the U.S. in March and early April of 2020 was undoubtedly instrumental in reducing number of infected cases [2]. However, due to pressure to relieve economic hardship and revive economies, imposed sanctions began to be lifted [3]. In the U.S., beginning in mid April of 2020, a number of states began lifting lockdown measures. This was followed by a dramatic increase in reported cases in many regions [4], which was then countered by returning to earlier stricter social distancing. After that a pattern of closing and opening up state economies ensued in reaction to perceived waves. Then in late December of 2020, COVID-19 vaccines became available to some of the U.S. population and a mass vaccination program began in earnest in 2021.

These factors have contributed to a complex dynamic process and because of this, many things remain unclear. For example, estimated fatality rates of early COVID-19 data have been reported to be 6–20% [5], but more recent data suggests far lower numbers. For the U.S., what is the rate? Has it changed, and if so when did this occur? Another matter are waves. Wave-like behavior of COVID-19 has been reported as matter of fact, but how many waves have occurred, when did they occur and what are their characteristics? A related issue is the metric used to measure fatality. We note that values 6–20% reported above are from calculations by David Baud and colleagues [5], which they referred to as a “mortality rate”, but which are technically the cumulative death rate, defined as the proportion of a group dying within a specified time interval [6]. A more widely adopted measure is case fatality rate, defined as the proportion of deaths due to a specific disease over total number of diseased cases relative to length of time. This work estimates a related value, the case fatality risk, abbreviated here as CFR, and defined as the probability of death for an infectious case. Here the term risk is used in place of rate, as rate refers to a specific time period, whereas risk refers to the probability of an adverse outcome [7]. The CFR is easily understood: *for an individual with COVID-19, what is the probability they will die?*

A widely used tool to study dynamics of an infectious disease are predictive epidemiological models [8–16]. One of the most commonly used of these is the SIR compartmental model [17]. This characterizes individuals of a population in terms of three stages of infection: susceptible, infected and recovered. Extensions to the basic SIR model to include other stages have been considered for COVID-19 [18–22]. An implicit assumption of compartmental models is an exponentially distributed infection time. Under this framework, most of the infected are assumed to recover or die early in the infection duration, which may not conform to observed COVID-19 survival behavior [23–25]. To overcome this, extensions to SIR rely on multiple stages using mathematical models with additional parameters; however these can be difficult to estimate especially with limited data available in epidemic scenarios. There has been some work to more directly attack this issue by using non-exponential distributions [26]. Lofgren et al. [22] grouped patients into several stages based on type of exposure and patient risk for analysis of fecal microbiota transplantation data. The integro-differential equation formulation [27, 28] and the method of stages [29–31] have been used for measles. However, these require the distribution to be nonincreasing [27] or assume the mortality in the exposed and infectious classes is ignorable [28, 30, 31].

While there has been a substantial effort to extend compartmental models for more realistic analysis of infectious data, an overlooked approach is directly addressing the competing forces at play that “remove” an infectious individual from a susceptible population. Current models do not make a distinction for removal, but when an individual becomes infected with COVID-19, they are removed due to one of two conditions occurring: death or cure. Thus at any given time, there is an instantaneous risk of either dying or being cured for the infected individual. In survival analysis, this type of data is called competing risk data and there is a large literature that has been developed for addressing this.

We extend the classical epidemiological compartmental model by incorporating competing risks using flexible hazard models for cure and death that can be used with epidemiological data. This yields a continuous removal rate that is a function of time and allows for flexible modeling of the dynamic process. It also makes it possible to estimate survival parameters for the disease, such as the probability of dying from COVID-19 (the CFR).

**Fig 1:**
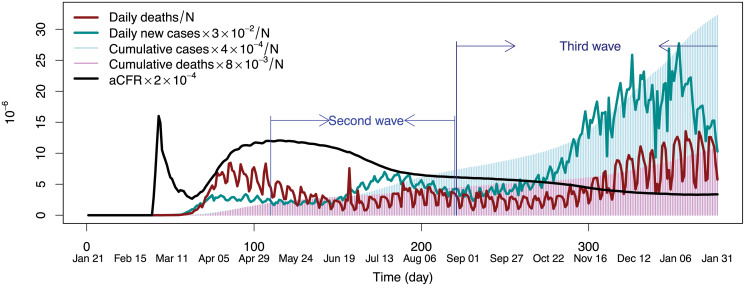
Statistics for COVID-19 pre-vaccination data in the U.S from January 21th, 2020 to February 1st, 2021. Note that values have been scaled in order to allow comparisons in one figure.

As motivation, consider Fig 1 which displays summary statistics for the U.S beginning from January 21th 2020, through to February 1st 2021. This will be referred to as pre-vaccination data as most of this data is prior to the large scale vaccination efforts of 2021. The figure highlights two waves, one being the time period of May 9 through August 27, which as will be explained is a period believed to characterize the second wave of the epidemic (the first wave being onset of the disease in early February). During this second wave period, there were 4,592,625 confirmed cases and 103,349 deaths from COVID-19 compared with 1,291,641 confirmed cases and 77,380 deaths recorded as of May 8th [32]. A unique pattern characterizing this second wave is a higher disease incidence rate combined with a lower apparent case fatality ratio (aCFR). The aCFR is defined as cumulative deaths due to the infection divided by cumulative confirmed cases and is a useful statistical quantity as it approximates the CFR. Fig 1 also highlights a third wave that follows completion of the second wave. Following a decrease in incidence rate for the second wave, it is characterized by an increasing incidence rate relative to daily deaths.

Using the competing risk compartmental model, we analyze pre-vaccination data using a four-parameter lognormal model in combination with a data driven contact rate. To tune parameters we make use of empirical data, a technique that has proven to be effective for describing the complex dynamics of COVID-19 [33]. Our analysis confirms the presence of the previously described waves and also chararacterizes their properties. We find that second and third waves have a significantly decreased CFR 1.7% compared with 8.5% of the first wave and also that the third wave has a longer period and a higher contact rate than previous waves. A what-if analysis which studies how dynamics change with parameters, reveals the importance of infectious time. This is crucial to understanding the effectiveness of vaccines. Extending the competing risk compartment model to include a vaccination component, we analyze 2021 vaccination data and find a fourth wave, however the effect of this wave is suppressed due to effectiveness of the vaccine. Parameters that play a crucial role in accurate modeling of this data are a lower cure time and significantly lower mortality rate for those vaccinated.

## Materials and methods

### Competing risk compartmental model

Let *S* denote number of susceptible, *I* the number of infected and *R* the number removed. We propose the following generalization of the SIR model [10]:

$$\begin{array}{cc} \frac{dS}{dt} & =-\frac{\beta (t)IS}{N}\\ \frac{dI}{dt} & =\frac{\beta (t)IS}{N}-\gamma \left(t\right)I\\ \frac{dR}{dt} & =\gamma (t)I. \end{array}$$

Here *N* = *S* + *I* + *R* is the total population which is assumed constant; thus the above set of equations reduces to two equations. The above generalizes the classical SIR model by allowing contact rate and removal rate to change with time. The value *β*(*t*) is the contact rate at time *t*, equal to average number of contacts per person per time multiplied by probability of disease contact between a susceptible and infectious case at time *t*. The function *γ*(*t*) denotes the removal rate at time *t*.

The removal rate is described as a function of *t* by utilizing a competing risk framework. We call this the Susceptible Infectious Cure Death (SICD) model. Let *X* be the continuous event time of an infected individual who either recovers from infection (is cured) or dies due to infection. The distributions for the two competing risks of *X* are specified using cause-specific hazard functions (one family that will be especially useful are lognormal distributed variables). Letting *h*_*C*_(*x*) and *h*_*D*_(*x*) denote the cause-specific hazards for cure and death (i.e. instantaneous risk of cure and death), we have the following key identity for the number removed (Theorem 1 in S1 Appendix),

$$\begin{array}{c} \frac{dR}{dt}=\gamma \left(t\right)I=\gamma_{C}\left(t\right)I+\gamma_{D}\left(t\right)I. \end{array}$$ (1)

The removal rate *γ*(*t*) equals *γ*_*C*_(*t*) + *γ*_*D*_(*t*) where *γ*_*C*_ and *γ*_*D*_ are the averaged *h*_*C*_ and *h*_*D*_ cause-specific hazards, averaged over length of time an infectious individual is infectious prior to *t*. This shows that the number of removed can be separated into number of cured and number dead in terms of the underlying hazards, which is an important feature exploited by our algorithm.

Identity (1) clarifies how the the choice of hazard effects the model. Consider the classical SIR model, which assumes *X* is exponentially distributed. Then *γ*(*t*) = *γ* is a constant function and the cause-specific hazards for cure and death are also constant functions, *h*_*C*_(*t*) = λ_*C*_, *h*_*D*_(*t*) = λ_*D*_ (Corollary 1 in S1 Appendix). Let *M*_death_ be the the limit of the cumulative incidence function for death, where the latter is defined as the probability of an infectious individual experiencing death by a specified time (Definition 1 in S1 Appendix). Then *M*_death_ equals the CFR and for the exponential model we have *M*_death_ = λ_*D*_/*γ*. Denoting the mean infectious period by $\bar{X}$, by the mean property of an exponential random variable, we have $\bar{X}=1/\gamma$. Therefore, $\lambda_{D}=M_{\text{death}}/\bar{X}$ and $\lambda_{C}=\left(1-M_{\text{death}}\right)/\bar{X}$, which shows that just fitting $\left(M_{\text{death}},\bar{X}\right)$ already uses up the two available degrees of freedom (λ_*C*_, λ_*D*_) for the model. This is one way to see why the classical model will be too inflexible for COVID-19 data.

### Discrete time model

The SICD model is numerically implemented using a discrete time algorithm that takes both time *t* and infectious duration *x* as discrete intervals so that the solution can be calculated iteratively (see Section S1.3 in S1 Appendix). Days *d* are used in place of *x* for infectious duration time. To indicate discrete time for *β* a subscript of *t* is used. Values *c*_*d*_ and *m*_*d*_ are discrete versions for the cure and death hazards *h*_*C*_ and *h*_*D*_.

The number of infectious cases *I*(*t*) on day *t* is $I\left(t\right)=\sum_{d=0}^{\infty }i\left(t,d\right)$ where *i*(*t*, 0) is the number of newly infected cases and *i*(*t*, *d*) is the number of infectious cases on day *t* who have been infected for *d* ≥ 1 days. The basic identity is

$$\begin{array}{c} N=I(t)+S(t)+R(t)=I(t)+S(t)+[C(t)+D(t)] \end{array}$$

where *R*(*t*) = *C*(*t*) + *D*(*t*) is the total number removed and *C*(*t*) and *D*(*t*) are the total of all cured and dead up to day *t*.

Moving from day *t* − 1 to day *t*, the *I*(*t* − 1) cases transmit disease to the susceptible cases at a discrete contact rate of *β*_*t*_. Consequently, the number of newly infected cases on day *t* is

$$\begin{array}{c} i\left(t,0\right)=\beta_{t}\frac{I(t-1)S(t-1)}{N}. \end{array}$$

**Fig 2:**
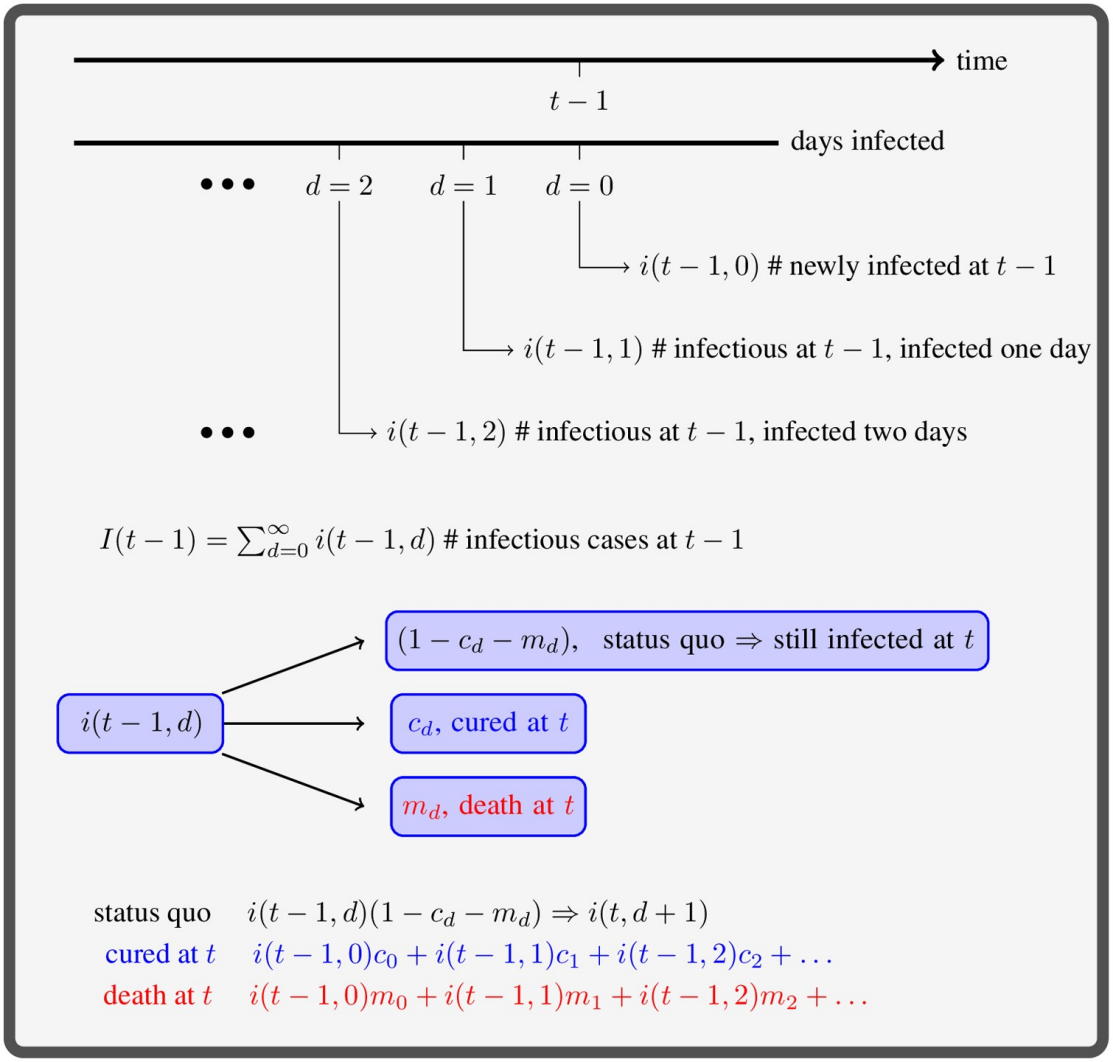
Infectious cases at time *t* − 1 who have been infected *d* days, *i*(*t* − 1, *d*), have three possible outcomes at time *t*. An infectious case is either cured, they die, or they remain infectious, with rates *c*_*d*_, *m*_*d*_, and 1 − *c*_*d*_ − *m*_*d*_, respectively.

There are three possible outcomes for the infectious cases on day *t* − 1 going to day *t*: cured, death, or infectious (status quo), with probabilities depending on infectious duration (Fig 2). For *i*(*t* − 1, *d*), the probability of cure is *c*_*d*_, the probability of death is *m*_*d*_, and the probability of remaining infectious is 1 − *c*_*d*_ − *m*_*d*_. The infectious cases, *i*(*t* − 1, *d*) × (1 − *c*_*d*_ − *m*_*d*_), will be counted as *i*(*t*, *d* + 1) on day *t* because their infectious duration increases one day, i.e. *i*(*t*, *d* + 1) = *i*(*t* − 1, *d*)(1 − *c*_*d*_ − *m*_*d*_). The cured cases, *i*(*t* − 1, *d*)*c*_*d*_, and deaths, *i*(*t* − 1, *d*)*m*_*d*_, are counted towards daily cured and deaths on day *t*, yielding

$$\begin{array}{c} \frac{dR}{dt}≍R\left(t\right)-R\left(t-1\right)=\sum_{d=0}^{\infty }i\left(t-1,d\right)c_{d}+\sum_{d=0}^{\infty }i\left(t-1,d\right)m_{d}. \end{array}$$

Hence, solutions for $I\left(t\right)=\sum_{d=0}^{\infty }i\left(t,d\right)$, *R*(*t*), and *S*(*t*) = *N* − *I*(*t*) − *R*(*t*) can be obtained once we are given values ${\left\{\beta_{t}\right\}}_{1}^{T_{\max}}$, ${\left\{i\left(0,d\right)\right\}}_{0}^{M}$, and $\left({\left\{c_{d}\right\}}_{0}^{M},{\left\{m_{d}\right\}}_{0}^{M}\right)$; the latter are conditional cure and death rates obtained from the discretized hazards for *h*_*C*_ and *h*_*D*_. Here *M* is a large number such that *i*(*t*, *d*) can be assumed to be zero for *d* > *M*; thus sums are constrained to *M* terms. The value *T*_max_ equals maximum number of days under study.

### Pre-vaccination model parameters and identification and observability

The discrete time algorithm was applied to New York Times COVID-19 data for the U.S. from January 21, 2020 to February 1, 2021 [32]. Population size was *N* = 325,217,163 equal to the sum of populations for the states and regions reported by the New York Times. Values were intialized using *i*(0, 0) = 1, and *D*(0) = *C*(0) = *i*(0, 1) = *i*(0, 2) = ⋯ = *i*(0, *M*) = 0. A value of *T*_max_ = *M* = 377 was used for the time window. Mean infectious duration was set to $\bar{X}=29$ days: 14 or more days to develop symptoms [34], 7 days of moving average of the interval from symptom onset to isolation in hospital or quarantine [35], and 8 days from hospital admission to mortality or discharge (the average of 7 days for mortality and 9 for discharge) [23].

For exponential hazards, corresponding to the classical SIR model, parameters were set to $\lambda_{C}=\left(1-M_{\text{death}}\right)/\bar{X}$ and $\lambda_{D}=M_{\text{death}}/\bar{X}$ (for the first wave in Fig 1 with 8.5% CFR this is λ_*C*_ = 0.032 and λ_*D*_ = 2.93 × 10^−3^). Lognormal cause-specific hazards were set to parameter values (*μ*_*C*_, *σ*_*C*_) for cure and (*μ*_*D*_, *σ*_*D*_) for death, where *μ*_*C*_ = 3.506, *σ*_*C*_ = 0.51, *μ*_*D*_ = 3.8, and *σ*_*D*_ = 0.91 (Section S2 in S1 Appendix). Regarding the issue of identifiability and observability, the classical SIR model is structurally identifiable with observable state *S*(*t*) when either *I*(*t*) or cummulative incidence data is used for the directly measured state [36]. These results continue to hold if the removal rate is a continuous time-varying function (see Model 6 from the S1 Appendix of [37]). Thus the SICD is identifiable with observable state *S*(*t*). Later we will investigate the issue of practical identifiability for the SICD model.

### Data-driven time varying contact rate

**Fig 3:**
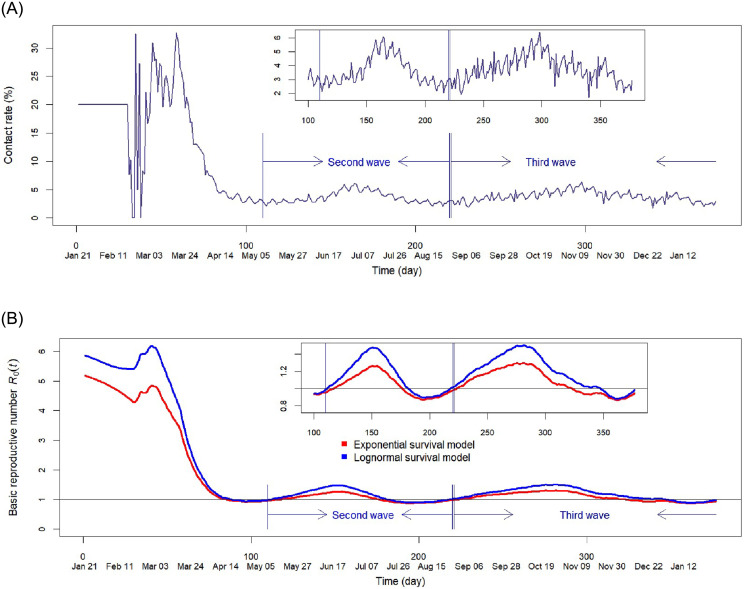
Pre-vaccination data. (A) Contact rate calculated as the fraction of new infected on day *t* in the total infected during day *t* − 29 through day *t* and set to 0.2 for the first 30 days. (B) Reproduction number *R*_0_(*t*) assuming data driven contact rate for exponential model (classical SIR model) and lognormal model where CFR is 8.5% for the first wave and 1.7% thereafter.

Data driven values were used for the discrete time contact rates *β*_*t*_ and set using the following approach. Let $I_{t}^{\text{new}}$ denote the observed number of newly infected cases on day *t*. Because cases reported before $\bar{X}$ days are typically either cured or dead, the discrete contact rate *β*_*t*_ was estimated by the following: $\beta_{t}=I_{t}^{\text{new}}/\sum_{\left(s=t-\bar{X}\right)}^{t}I_{s}^{\text{new}}$ (Fig 3A).

The basic reproduction number, denoted as *R*_0_, equals expected number of infections arising due to contact with a positive case in a population where all individuals are susceptible to infection [38–40]. With a time varying contact rate, the reproduction number generalizes to

$$\begin{array}{c} R_{0}\left(t\right)=\int_{0}^{\infty }[\int_{t}^{t+x}\beta \left(s\right)\;ds]\;f_{X}\left(x\right)\;dx \end{array}$$

which equals total (integrated) contact rate for an individual infected at *t*, averaged with respect to length of infectious duration, *X*. A discrete estimate for *R*_0_(*t*) was obtained by discrete integral calculus over *β*_*t*_ where the density *f*_*X*_ for *X* is obtained from the discrete survival model (Section S1.3 in S1 Appendix). Note if contact rate is constant *β*_*t*_ = *β*, then $R_{0}=\beta \bar{X}$ which equals *β*/*γ* for the classical compartmental model [8, 10, 17, 41].

## Results

### Survival model specification using a simulation with fixed contact rate

We first examined the effect of survival model specification on disease spread using a simulation under a fixed contact rate. Using the discrete time algorithm, we compared performance of exponential and lognormal distributed models (specified in S1 and S2 Figs in S1 Appendix). The contact rate was set to a constant *β* = 0.2. Survival models were calibrated to have equal infectious duration and CFR. We observe significantly different behavior for the models. For the lognormal, peak of daily deaths occurs after peak of infectious cases, while for the exponential, peaks occur at the same time. This delay pattern for the lognormal is more realistic. Death and infectious peaks are highest for the lognormal (S3 and S4 Figs in S1 Appendix; S1 Video). Therefore, even with the same mean infectious duration and CFR, the type of survival model yields substantial difference in disease spread.

### Analysis of pre-vaccination data

U.S. pre-vaccination data was then analyzed using the fully time varying SICD model, which included the time-varying data-driven discrete time contact rate *β*_*t*_ described earlier. The latter is shown in Fig 3A. Models were fit with CFR set to 8.5% so as to generate realistic proportion of daily deaths over daily infected. All models are able to reasonably approximate aCFR for the first wave defined as COVID-19 prior to May 9th (S5 Fig in S1 Appendix). However, all models overestimate aCDR after first wave.

Given this overestimation, we hypothesized that CFR must have decreased after easing of lockdown measures. To investigate this, models were re-estimated under previous parameter values but assuming a decreased CFR of 1.7% for the period following the first wave. To estimate values, the discrete time algorithm was applied to data in the period defined by the first wave using a CFR of 8.5% and then separately to post-first wave data using a CFR of 1.7%.

**Fig 4:**
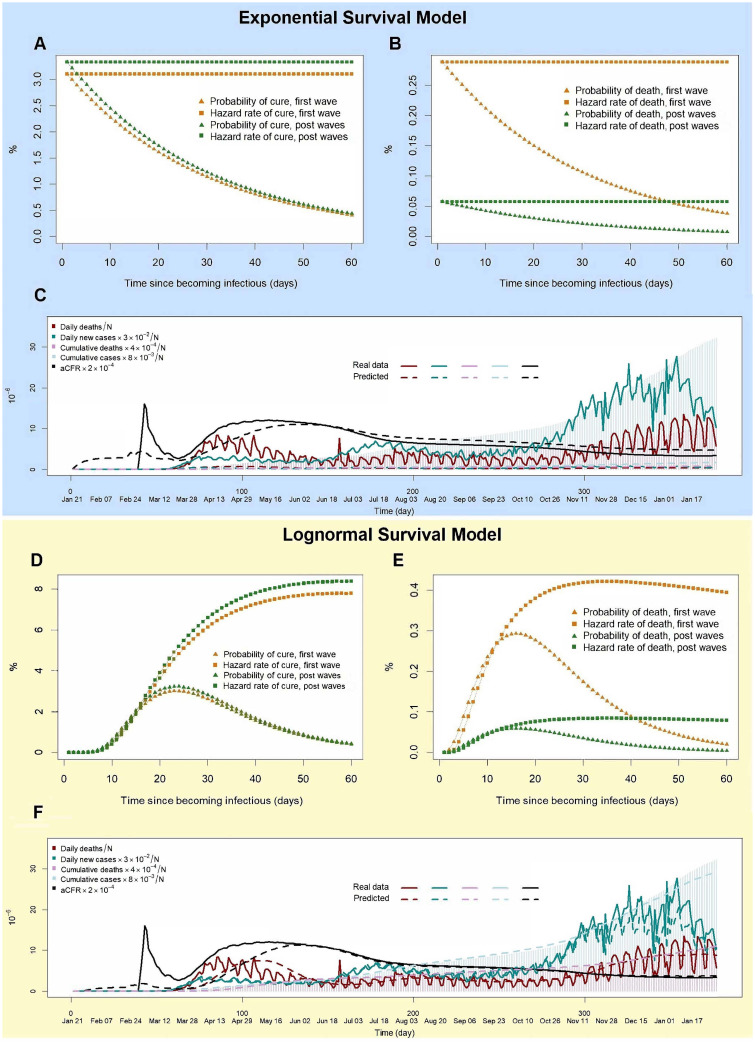
COVID-19 analysis of pre-vaccination data using exponential model (classical SIR model) and lognormal model assuming CFR is 8.5% for the first wave and 1.7% thereafter. (A,B) Probability and hazard values for cure and death for exponential model. (C) Estimated aCFR, cumulative and daily infected cases using exponential model compared to observed values. (D,E) Probability and hazard values for cure and death for lognormal model. (F) Estimated aCFR, cumulative and daily infected cases using lognormal model compared to observed values.

Fig 3B displays estimated *R*_0_(*t*) under the above settings. Both lognormal and exponential models have *R*_0_(*t*) that begin approximately at 1.0 at the start of second wave. Values increase and decrease completing a full period returning to the starting value of 1.0. This provides further confirmation of a second wave distinct from previous values (a similar pattern is observed for the third wave although it has a longer period). Under this adjusted decreased CFR, the lognormal model is now able to accurately approximate observed values of aCFR, daily infected and deaths, over all periods of the data (Fig 4F, S2 Video). The exponential model (Fig 4C) is however unreliable and underestimates both number of daily deaths and infectious cases. This is due to the faster recovery rate imposed by the exponential distribution assumption. A bimodal lognormal distribution included in our comparison also performs poorly (S6C Fig in S1 Appendix). From Fig 4 it can be concluded the lognormal model is the most accurate and realistic model. Only this model will be considered for the remaining analysis.

### What-if analysis: Practical identifiability

Values for the bivariate process of daily new infected cases and daily deaths, denoted by $\left(\dot{I}\left(t\right),\dot{D}\left(t\right)\right)$, were estimated under different parameters for the SICD lognormal model as a means to assess practical identifiability. The lognormal model is dependent on four parameters, and these are tuned according to desired values for $\bar{X}$ and *M*_death_. For this reason, the structural parameter of interest *θ* for practical identifiability can be considered to be $\left(\bar{X},M_{\text{death}}\right)$. Thus practical identifiability for the SICD model is assessed by considering $g\left(t;\theta \right)=(\dot{I}\left(t\right),\dot{D}\left(t\right);\theta )$. Practical identifiability means that *g*(*t*;*θ*), number of daily new infected cases and daily deaths, is identified as a function of *θ*, i.e. mean infectious duration and CFR.

**Fig 5:**
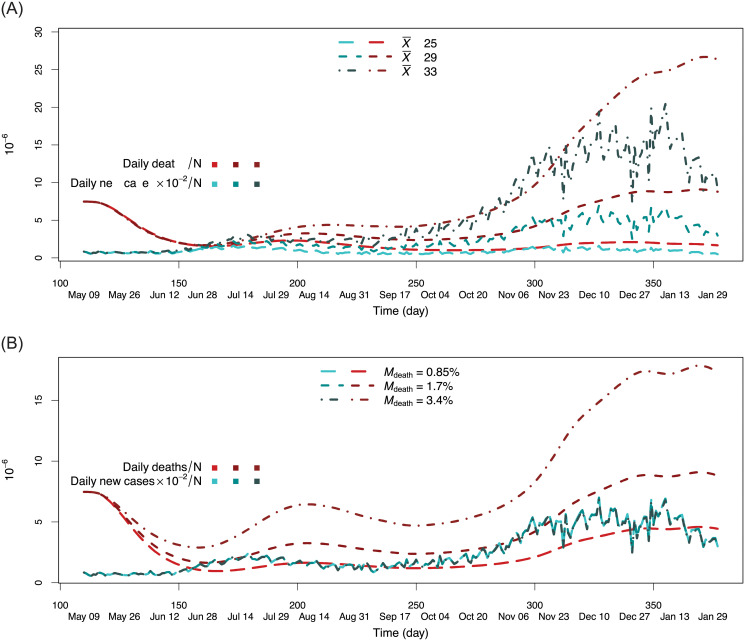
What-if practical identifiability analysis of SICD lognormal model. Estimated values for daily infected and deaths are given by for different $\bar{X}$ and *M*_death_ values (i.e. what if $\bar{X}$ equals this and what if *M*_death_ equals this): (A) $\bar{X}=25,29,33$ (B) *M*_death_ = 0.85%, 1.7% and 3.4%.

Fig 5(A) displays estimated daily new infected cases and daily deaths under different settings for $\bar{X}$. Three values were used, $\bar{X}=25,29,33$, with all other parameters for the SICD lognormal model set to previous values. Fig 5(B) displays estimated values using *M*_death_ = 0.85%, 1.7% and 3.4%. All other parameters were set as before. In both figures at time zero, *g*(*t*;*θ*) is the same, however as *t* increases the bivariate process *g*(*t*, *θ*) is identified in *θ*. In particular, note that in Fig 5(B) although number of daily new cases is not affected by varying *M*_death_ (which is to be expected), the number of daily deaths changes quite dramatically. Thus when taken together as a bivariate proceess, this shows *g*(*t*;*θ*) is identified.

This what-if analysis also revealed the importance of the two parameters studied. To put some numbers to this, assuming $\bar{X}=29$ days, we estimated 23,914,491 cumulative infected cases with 18,848,443 of these being cured after 377 days. If infectious time is shortened so that $\bar{X}=25$, we estimate only 9,326,640 cumulative infected case of which 8,357,707 are cured. This also results in the number of cumulative deaths being reduced from 452,522 to 248,085. This demonstrates the importance cure time has on the disease.

### 2021 vaccination data and extension to a vaccine compartment

Although from Fig 4 the estimated deaths and aCFR are very close to true recorded numbers for 2020, estimated infected cases in early 2021 were found to be underestimated. There is a second peak around January 6th, 2021 with the largest number of daily new infected cases. This is likely due to the especially high contact rate during the holidays.

Therefore, between December 20th, 2020 and January 5th, 2021, the contact rate was increased to *β*_*t*_ ← 1.35 × *β*_*t*_ (335 ≤ *t* ≤ 351) which allows the estimated infected cases to match the observed cases. However, deaths after 2021 are overestimated, which indicates that vaccines that became available in December 2020 must have improved survival. Therefore to address this, we extended the SICD model to include a vaccination compartment. This new model is referred to as the Susceptible Infectious Vaccinated Cure Death Immune (SIVCDI) model.

**Fig 6:**
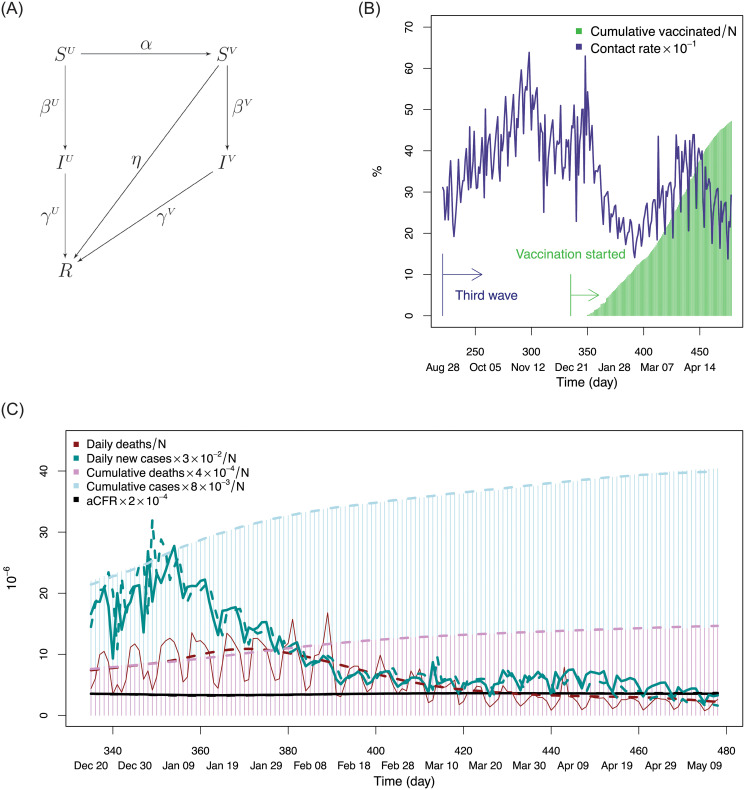
Vaccination model results. (A) Graphical representation of SIVCDI model. Removed status includes cured, dead, and immune from vaccination. (B) Contact rate and percentage of vaccinated population. Contact rate shows presence of a small wave after February 2021, which could be a potential fourth wave, but because of the success of vaccination, the fourth wave contact rate is much lower than the third wave contact rate. (C) Results of the data driven SIVCDI model from December 20th, 2020 to May 11th, 2021, where dashed lines are predicted values and solid lines are observed values.

In this extension, the suspectible and infectious are separated into two groups. Unvaccinated susceptible cases are denoted by *S*^*U*^ and vaccinated susceptible cases are denoted by *S*^*V*^, with *S* = *S*^*U*^ + *S*^*V*^ as their sum. Likewise, unvaccinated infectious cases are denoted as *I*^*U*^ and vaccinated infectious are denoted as *I*^*V*^, with *I* = *I*^*U*^ + *I*^*V*^ as their sum. Associated parameters are also separated into two groups. The SIVCDI model is as follows (see Fig 6(A)):

$$\begin{array}{cc} \frac{dS^{U}}{dt} & =-\alpha \left(t\right)S^{U}-\frac{\beta^{U}\left(t\right)IS^{U}}{N}\\ \frac{dS^{V}}{dt} & =\alpha \left(t\right)S^{U}-\frac{\beta^{V}\left(t\right)IS^{V}}{N}-\eta \left(t\right)S^{V}\\ \frac{dI^{U}}{dt} & =\frac{\beta^{U}\left(t\right)IS^{U}}{N}-\gamma^{U}\left(t\right)I^{U}\\ \frac{dI^{V}}{dt} & =\frac{\beta^{V}\left(t\right)IS^{V}}{N}-\gamma^{V}\left(t\right)I^{V}\\ \frac{dR}{dt} & =\gamma^{U}\left(t\right)I^{U}+\gamma^{V}\left(t\right)I^{V}+\eta \left(t\right)S^{V}. \end{array}$$

Here *α*(*t*) is the vaccination rate at time *t*, *β*^*U*^ is the effective contact rate for unvaccinated cases at time *t*, equal to average number of contacts per person per time multiplied by probability of disease transmission between a unvaccinated susceptible and infectious case at time *t*, *β*^*V*^ is the contact rate between a vaccinated susceptible case and infectious case at time *t*, *γ*^*U*^(*t*) denotes the removal rate for unvaccinated infectious cases at time *t*, *γ*^*V*^(*t*) is the removal rate for the vaccinated infectious cases and *η*(*t*) is the immune rate equal to percentage of vaccinated individuals who become immune to the disease. As before, the sample size *N* is fixed and the above equations can be reduced by one using *N* = *S*^*U*^ + *S*^*V*^ + *I* + *R*.

The analysis used 2020 and 2021 data (*t* ≥ 335) from the New York Times COVID-19 repository [32], combined with publicly available vaccination data [42] recording number of vaccines administered per day. Calculations were based on a discrete time algorithm (Section S4.1 in S1 Appendix). Previously for the SICD model, *X* equals the continuous event time of an infected individual who either recovers from infection or dies due to infection. With the SIVCDI model, *X* becomes *X*_*U*_ and we add a new continuous variable *X*_*V*_, defined as the event time that an infected vaccinated individual either recovers or dies. Related to *X*_*V*_ is is another new variable *E*_*V*_, defined as the event time for a vaccinated individual who becomes immune. We define the mean immunity period as ${\bar{E}}_{V}$, equal to average number of days vaccinated individuals who become immune require to develop immunity. This was set to ${\bar{E}}_{V}=30$ days but results were fairly robust to its choice. Based on the previous analysis showing benefit of improved infectious time, the average cure time for infected vaccinated individuals was set to 25 days. Mortality rates for vaccinated individuals has been observed to be extremely low, therefore the death rate for this group was set to *M*_death_ = .001%. Contact rates *β*^*U*^ and *β*^*V*^ were estimated using data from published studies of vaccine efficacy (Section S4.2 in S1 Appendix). All other parameters including parameters for the unvaccinated infecious cases were set as before for the pre-vaccination data analysis. The results for the data driven SIVCDI model are given in Fig 6(C). As can be seen, predicted values are near identical to observed values. The contact rate is displayed in Fig 6(B) and shows a reduced wave after February 2021, which could be a potential fourth wave. Its value is reduced relative to the third contact rate wave due to success of the vaccination.

## Discussion

Time to event subject to competing risks is a branch of survival analysis called competing risk analysis. While clinical data is often used in medicine for such analyses, this type of setting is very different than the setting where a general population is exposed to a rapidly manifesting infectious disease. For clinical data, the initial time is often recorded as time of hospitalization, or diagnosis for a specific stage of disease development [43, 44], rather than onset of disease, which is what is needed to model infectious duration of a disease. Also, clinical settings often target specific populations and therefore their results may not translate to general populations. With this in mind, we developed our compartmental model using a competing risk framework (SICD model) where survival model parameters can be determined from aggregated population level epidemiological data. This is the type of data one typically has to work with when an infectious disease strikes.

The advantages of a flexible competing risk framework are clear when compared with the classical SIR model which assumes a constant hazard. In infectious diseases, the hazard rate at the beginning of disease development is often very low with increasing values later in time. The lognormal distribution [45, 46] is well suited for this type of modeling as it accomodates hazards that can increase and decrease [47]. The distribution is conveniently specified by two parameters, and these can easily be tuned so that peak of death occurs after peak of infectious cases, which is suggested by COVID-19 data. Separate lognormal parameter values are used for cure and death as the magnitude of risk for cure is much larger than death.

This work has shown using just four parameters, that the lognormal distribution can accurately model COVID-19 pre-vaccination data when used in combination with a data-driven dynamic contact rate. The model was able to accurately estimate dynamic values like the aCFR and daily number of infected and deaths, but also at the same time provide estimated values for key survival parameters such as the CFR. Although the lognormal was used exclusively here, other distributions could also be used; for example, a promising choice might be the Erlang distribution [30]. However the lognormal proved robust in our experimentation. Also because of certain numerical simplifications that occur for this distribution (Section S2.1 in S1 Appendix), we found it very convenient for numerical calculations.

The SICD competing risk model was extended by adding a vaccination compartment and applied to 2021 vaccination data. Parameters for the extended SIVCDI model were separated into the two groups of vaccinated and unvaccinated and were relatively easy to specify using published studies and publicly available data. As was shown, the SIVCDI model could accurately fit the 2021 observed data. As has been noted, parameters that played a crucial role in this accurate modeling were a lower cure time and a lower mortality rate for those vaccinated. Both adjustments are quite realistic given the importance of a reduced cure time suggested by a what-if analysis and the wide spread consensus that vaccination signficantly reduces mortality.

## Supporting information

S1 Appendix (ZIP)

S1 Video Comparison of three survival models with 29-day mean infectious duration, 8.5% morality and 0.2 fixed contact rate. (MP4)

S2 Video Analysis of COVID-19 U.S. data with with 29-day mean infectious duration and data-driven contact rate. (MP4)

S1 FigComparison of three different survival models where all models have identical mean infectious period $\bar{X}=29$ and mortality rate *M*_death_ = 8.5%.Shown are CCDF $\bar{F}(t)$ (black), CIF for cure *F*_1_(*t*) (orange) and CIF for death *F*_2_(*t*) (green). (A) Scenario I uses an exponential distribution, which is equivalent to the classical SIR model by Corollary 1 of Appendix. (B) Scenario II uses a lognormal distribution. (C) Scenario III uses a bimodal lognormal distribution.(PDF)

S2 Fig Discrete time survival values for scenario I (red), II (blue) and III (purple). (A) Discrete time pseudo-densities for cure. Most infections recover at the beginning in scenario I; around 15-40 days in scenario II; and either within 17 days, or around 25 to 55 days, in scenario III. (B) Discrete time pseudo-densities for death. Most deaths occur at the beginning in scenario I and around 15-40 days in scenarios II and III. (C) Discrete time hazard rates for cure. Scenario I has constant hazard whereas scenarios II and III assume hazards that initially increase and then decrease. Scenario III assumes a bimodal shape for the cure hazard. (D) Discrete time hazard rates for death.
(PDF)

S3 FigDiscrete time SIR models assuming a constant contact rate *β*(*t*) = 0.2.Infectious cases *I*(*t*) (black), daily cured cases $\dot{C}\left(t\right)$ (orange) and daily deaths $\dot{D}\left(t\right)$ (green) are displayed as percentage of total population. Daily cured and deaths being much smaller than *I*(*t*) are multiplied by 30 and 200. (A) In scenario I, all values have the same trend and peak at the same time. (B) In scenario II, daily deaths peak after infectious cases, which is more realistic. (C) In scenario III, deaths also peak after infectious cases, but daily cured has two waves due to the bimodal distribution assumption.(PDF)

S4 Fig Comparison of discrete time SIR models assuming a constant contact rate *β*(*t*) = 0.2. (A) Daily cured. (B) Daily deaths. (C) aCDR. (D) Infectious cases as percentage of population, *I*(*t*)/*N*. Values of aCDR should be very low at onset of disease due to few cures and death occuring immediately after infection. Therefore, scenario I is unrealistic.
(PDF)

S5 Fig Analysis of COVID-19 pre-vaccination data assuming a constant mortality rate for first and subsequent waves. Scenario II is best at estimating daily new cases. However, aCDR and daily deaths are overestimated after first wave, thus suggesting a lower mortality for post-first wave data.
(PDf)

S6 Fig Analysis of COVID-19 pre-vaccination data assuming a lower mortality rate for second and subsequent waves. (A) Basic reproduction number *R*_0_(*t*); note its values are much smaller for Scenarios I and III than II. (B) Even though I and III have similar *R*_0_(*t*) profiles, estimated values for daily new infections and deaths are different. (C) Bimodal lognormal distribution continues to perform poorly even under assumption of lower mortality for post-first wave data.
(ZIP)
