# Supplementary material for: Cure and death play a role in understanding dynamics for COVID-19: Data-driven competing risk compartmental models, with and without vaccination
Source: PLoS One. 2021 Jul 15;16(7):e0254397. doi: 10.1371/journal.pone.0254397 (PMC8282006; doi:10.1371/journal.pone.0254397)
Supplement: S1 Appendix — (ZIP) [file pone.0254397.s001.zip › plos_supporting_info_2021_07_02.pdf]

Supplementary Materials for

Cure and death play a role in understanding dynamics for COVID-19: data-driven competing risk compartmental models, with and without vaccination

Min Lu[1], Hemant Ishwaran[1¤*]

[1]Department of Public Health Sciences, Miller School of Medicine, University of Miami, Miami, FL, USA

¤Current Address: Department of Public Health Sciences, Miller School of Medicine, University of Miami, Don Soffer Clinical Research Center, 1120 NW 14th Street, Miami, FL 33136, USA
* Corresponding author
E-mail: hemant.ishwaran@gmail.com (HI)

S1 Susceptible Infectious Cure Death (SICD) model

We generalize the classical SIR model [32] via the following set of ordinary differential equations:

$$\begin{aligned}\frac{dS}{dt} &= -\frac{\beta(t)IS}{N}\\ \frac{dI}{dt} &= \frac{\beta(t)IS}{N} - \gamma(t)I\\ \frac{dR}{dt} &= \gamma(t)I,\end{aligned} \tag{S1}$$

where S is number of susceptible, I is number of infected, R is number of removed (either died or recovered), $N = S + I + R$ is the total population which is assumed to be fixed, $\beta(t)$ is the contact rate at time t, equal to average number of contacts per person per time multiplied by probability of disease transmission between a susceptible and infectious case at time t, and $\gamma(t)$ denotes the removal rate at time t. In the classical SIR model, $\beta(t)$ and $\gamma(t)$ are assumed to be constants, $\beta(t) = \beta$ and $\gamma(t) = \gamma$ [32–35].

We extend [S1] to incorporate flexible survival models for length of infectious time. We refer to this as the Susceptible Infectious Cure Death (SICD) model. Let X be the continuous event time of an infected individual who either recovers from infection or dies due to infection. Let $\delta \in \{1, 2\}$ be the indicator recording which event occurs: $\delta = 1$ denotes cure (recovery); $\delta = 2$ denotes death. Let $F_j(x) = \mathbb{P}\{X \le x, \delta = j\}$ be the cumulative incidence function (CIF) defined as the probability of experiencing the event $j = 1, 2$ by time x. The CIF is related to the complementary cumulative distribution function (CCDF) $\bar{F}(x) = \mathbb{P}\{X \ge x\}$ by the identity

$$\begin{aligned}\bar{F}(x) &= 1 - \mathbb{P}\{X \le x\}\\ &= 1 - [\mathbb{P}\{X \le x, \delta = 1\} + \mathbb{P}\{X \le x, \delta = 2\}]\\ &= 1 - F_1(x) - F_2(x).\end{aligned}$$

Observe that x refers to the time passed in an infectious period for an infected case, not the time t passed since an epidemic outbreak.

An important quantity is the cause-specifc hazard function. The cause-specific hazard h_1 for cure and h_2 for death are

$$h_j(x) = \lim_{\Delta \downarrow 0} \left\{ \frac{\mathbb{P}\{x - \Delta \le X \le x + \Delta, \delta = j | X \ge x\}}{\Delta} \right\} = \frac{f_j(x)}{\bar{F}(x)}, \qquad j = 1, 2$$

where $f_j(x) = dF_j(x)/dx$ is the pseudo-density function where $f_1 + f_2 = f$ and f is the density of X. The following result characterizes the removal rate $\gamma(t)$ in terms of the cause-specific hazard functions.

Theorem 1. *Let $X(t) \in [0, \infty)$ be the length of time an individual has been infected at time t and denote its probability density function by $f_{X(t)}(x)$ where $\int_0^\infty f_{X(t)}(x)dx = 1$. Then*

$$\gamma(t) = \int_0^\infty \big[h_1(x) + h_2(x)\big] f_{X(t)}(x)\, dx. \tag{S2}$$

Proof. The number of newly removed cases at time t equals

$$\begin{aligned}
\frac{dR}{dt} &= I \cdot \lim_{\Delta \downarrow 0} \left\{ \frac{\mathbb{P}\{X(t) - \Delta \le X \le X(t) + \Delta\}}{\Delta} \right\} \\
&= I \cdot \lim_{\Delta \downarrow 0} \left\{ \Delta^{-1} \int_0^\infty \mathbb{P}\big\{x - \Delta \le X \le x - \Delta | X(t) = x\big\} f_{X(t)}(x)\, dx \right\} \\
&= I \cdot \lim_{\Delta \downarrow 0} \left\{ \Delta^{-1} \int_0^\infty \mathbb{P}\big\{x - \Delta \le X \le x - \Delta | X \ge x\big\} f_{X(t)}(x)\, dx \right\} \\
&= I \cdot \int_0^\infty \frac{f(x)}{\bar{F}(x)} f_{X(t)}(x)\, dx \\
&= I \cdot \int_0^\infty \frac{f_1(x) + f_2(x)}{\bar{F}(x)} f_{X(t)}(x)\, dx.
\end{aligned}$$

Comparing this with $dR/dt = I\gamma(t)$ in [S1] establishes [S2] as $h_j = f_j/\bar{F}$ by definition. □

Corollary 1. *If the cause-specific incidence functions are specified exponentially by*

$$F_1(x) = \frac{\lambda_1}{\gamma}(1 - e^{-\gamma x}) \quad \textit{and} \quad F_2(x) = \frac{\lambda_2}{\gamma}(1 - e^{-\gamma x}), \tag{S3}$$

where $\lambda_1 + \lambda_2 = \gamma$, then X is exponentially distributed with mean $1/\gamma$ and the cause-specific hazards are constants λ_1 and λ_2 and $\gamma(t) = \gamma$.

Proof. By [S3], the pseudo-density functions are

$$f_1(x) = \frac{dF_1(x)}{dx} = \lambda_1 e^{-\gamma x} \quad \text{and} \quad f_2(x) = \frac{dF_2(x)}{dx} = \lambda_2 e^{-\gamma x}.$$

Because $\lambda_1 + \lambda_2 = \gamma$,

$$\bar{F}(x) = 1 - F_1(x) - F_2(x) = 1 - \frac{\lambda_1 + \lambda_2}{\gamma}(1 - e^{-\gamma x}) = e^{-\gamma x}.$$

Therefore X is exponentially distributed with mean $1/\gamma$. Further, the cause-specific hazards are

$$h_1(x) = \frac{f_1(x)}{\bar{F}(x)} = \lambda_1 \quad \text{and} \quad h_2(x) = \frac{f_2(x)}{\bar{F}(x)} = \lambda_2.$$

Hence by Theorem 1, $\gamma(t) = \int_0^\infty [\lambda_1 + \lambda_2] f_{X(t)}(x)\, dx = \gamma \int_0^\infty f_{X(t)}(x)\, dx = \gamma$. □

Corollary 1 shows that the classical SIR model assumes an exponential distribution for X and constant cause-specific hazards. This has many implications: in particular the assumption of a fixed constant hazard process for cure and death will not be flexible enough for COVID-19 modeling. The exponential assumption also forces the value of mortality to a specific value.

Definition 1. *Define the mortality rate of the disease, denoted by M_{death}, as the limit of the CIF for the cause-specific death event: $M_{\text{death}} = \lim_{x \to \infty} F_2(x)$.*

Thus under the classical SIR model, the mortality rate is a fixed ratio of the death and cure rates specified by:

$$M_{\text{death}} = \lim_{x\to\infty} F_2(x) = \lim_{x\to\infty} \frac{\lambda_2}{\gamma}(1-e^{-\gamma x}) = \frac{\lambda_2}{\lambda_1+\lambda_2}. \tag{S4}$$

Theorem 1 allows us to utilize flexible survival models for $\gamma(t)$ using hazard functions that are more realistic than the exponential. Section S1.1 formalizes how this is accomplished through a competing risk formulation. We note that although it is almost impossible to identify $f_{X(t)}(x)$ in a closed format, which is required in the specification of $\gamma(t)$ and $dR/dt = I\gamma(t)$, we introduce a discrete time algorithm to compute $I \cdot f_{X(t)}(x)$ numerically in Section 1S1.3. The key step is to count cure and death separately when calculating removed cases, which holds by Theorem 1 due to

$$\frac{dR}{dt} = I\int_0^\infty \big[h_1(x)+h_2(x)\big] f_{X(t)}(x)\,dx = I\int_0^\infty h_1(x) f_{X(t)}(x)\,dx + I\int_0^\infty h_2(x) f_{X(t)}(x)\,dx.$$

Although this type of numerical solution will not provide analytical results for equilibrium stability and asymptotic behavior analysis, it is well suited for modeling discrete time data such as COVID-19.

S1.1 Reformulation of the CIF using competing risks

The CIF is a basic quantity in competing risks and plays a key role in calculations of [S1]. However, the CIF is not an easy or intuitive quantity to work with due to it being a complex function of the cause-specific hazards. This can be seen by the following argument. Recall $f_j = \bar{F}h_j$, and therefore

$$F_j(x) = \int_0^x f_j(s)\,ds = \int_0^x \bar{F}(s)h_j(s)\,ds = \int_0^x \bar{F}(s)\,dH_j(s) \tag{S5}$$

where $H_j(x) = \int_0^x h_j(s)ds$ is the cause-specific cumulative hazard function (CHF). By the mutual exclusiveness of competing risks, the hazard for X is $h(x) = h_1(x) + h_2(x)$. Because X is a continuous random variable, $\bar{F}(x) = \exp(-H(x))$ where $H(x) = \int_0^x h(s)ds$ is the CHF for X. Therefore,

$$F_j(x) = \int_0^x \exp\left(-\int_0^s \sum_{l=1}^2 h_l(u)\,du\right) dH_j(s) \tag{S6}$$

which is a complicated function involving both cause-specific hazards.

Now we describe the CIF in a different way. The reformulation will allow us to use hazard functions of our chosing, thus permitting flexible and intuitive modeling, but will also be done in such a manner so as to simplify downstream numerical calculations. Let X_j be a continuous random variable with hazard h_j for $j = 1, 2$. The key point here is that the hazard for X_j is selected to match the desired cause-specific hazard function, however X_j is an entirely theoretical construct that in no way is related to the true event time X. Let f_{T_j} and F_{T_j} be the density and cumulative distribution function (CDF) for X_j. Thus

$$h_j(x) = \frac{f_{T_j}(x)}{1 - F_{T_j}(x)} = \frac{f_{T_j}(x)}{\bar{F}_{T_j}(x)}$$

where $\bar{F}_{T_j}(x) = \exp(-H_j(x))$ is the CCDF for X_j. We can rewrite [S6] as

$$F_j(x) = \int_0^x \exp(-H_1(s))\exp(-H_2(s))\,dH_j(s)$$

and therefore

$$F_j(x) = \int_0^x \bar{F}_{T_1}(s)\bar{F}_{T_2}(s)h_j(s)\,ds = \int_0^x \bar{F}_{T_1}(s)\bar{F}_{T_2}(s)\frac{f_{T_j}(s)}{\bar{F}_{T_j}(s)}\,ds.$$

Cancelling the common value in numerator and denominator we obtain

$$F_1(x) = \int_0^x \bar{F}_{T_2}(s)\,dF_{T_1}(s),\;\; F_2(x) = \int_0^x \bar{F}_{T_1}(s)\,dF_{T_2}(s), \tag{S7}$$

which presents us with a useful characterization of the CIF that can be deployed in our algorithms. Notice also by [S5], the above implies

$$f_1(x) = \bar{F}_{T_2}(x)f_{T_1}(x) = \big[1-F_{T_2}(x)\big]f_{T_1}(x), \quad f_2(x) = \bar{F}_{T_1}(x)f_{T_2}(x) = \big[1-F_{T_1}(x)\big]f_{T_2}(x).$$

S1.2 Basic reproduction number

The basic reproduction number, denoted as R_0, equals expected number of infections arising due to contact with a positive case in a population where all individuals are susceptible to infection [36–38]. Because [S1] assumes a potentially non-constant contact rate $\beta(t)$, the reproduction number is allowed to change across time.

Definition 2. *Define the basic reproduction number at time t as expected number of cases arising due to contact with an individual who is infected at time t where all individuals are susceptible to infection:*

$$R_0(t) = \mathbb{E}\left[\int_t^{t+X} \beta(s)\,ds\right] = \int_0^\infty \left[\int_t^{t+x} \beta(s)\,ds\right]\big[f_1(x) + f_2(x)\big]\,dx. \tag{S8}$$

The length of infectious time X is a random variable. A useful way to summarize its value is by the mean which can be conveniently calculated using the CCDF [39].

Definition 3. *Define the mean infectious period, denoted by $\bar{X}$, as the expected value of infectious time:*

$$\bar{X} = \int_0^\infty xf(x)\,dx = \int_0^\infty \bar{F}(x)\,dx = \int_0^\infty \big[1 - F_1(x) - F_2(x)\big]dx. \tag{S9}$$

The inverse of the mean infectious time $1/\bar{X}$ represents a target value for assessing whether $R_0(t) < 1$ or $R_0(t) > 1$.

Theorem 2. *If $\beta(t)$ has an upper bound such that $\beta(t) \le 1/\bar{X}$, then $R_0(t) \le 1$.*

Proof.

$$R_0(t) = \mathbb{E}\left[\int_t^{t+X} \beta(s)\,ds\right] \le \frac{1}{\bar{X}} \cdot \mathbb{E}\left[\int_t^{t+X} ds\right] = \frac{1}{\bar{X}} \cdot \mathbb{E}[X] = 1.$$

□

Observe when $\beta(t) = \beta$ is a constant, as assumed by the classical SIR model, we have

$$R_0(t) = \mathbb{E}\left[\int_t^{t+X} \beta\,ds\right] = \beta \cdot \mathbb{E}\left[\int_t^{t+X} ds\right] = \beta \cdot \mathbb{E}[X] = \beta\bar{X} := R_0.$$

Therefore under the exponential model we have the following characterization of the basic reproduction number.

Theorem 3. *For the exponential model described in Corollary 1, the mean infectious period is*

$$\bar{X} = \int_0^\infty \big[1 - F_1(x) - F_2(x)\big]dx = \int_0^\infty e^{-\gamma x}dx = 1/\gamma.$$

Thus $R_0 = \beta\bar{X} = \beta/\gamma$ *and* $R_0 < 1$ *if and only if* $\beta < \gamma = 1/\bar{X}$.

For COVID-19, we will provide evidence that $\beta(t)$ is non-constant due to fluctuating containment measures and social distancing. Therefore this makes $\beta(t)$ of critical importance when assessing whether $R_0(t) < 1$.

S1.3 Algorithm for a discrete time SICD model

It is difficult to solve [S1] directly for an arbitrary $\gamma(t)$. Therefore, we take a discrete time approach [40] and describe an algorithm that takes both time t and infectious duration x as discrete intervals. This will allow the solution to be calculated iteratively. Denote $I(t)$ as the number of infectious cases on day t and define

$$i(t,d) = I\int_{d-1}^{d} f_{X(t)}(x)\,dx, \qquad d = 1,2,\ldots,M,$$

as the number of infectious cases at time t who have been infected for $x = d$ days. Here M is a preset number chosen large enough so that $i(t, M+1) := I\int_M^\infty f_{X(t)}(x)dx$ can safely be assumed to equal zero. Let $i(t,0)$ be the number of newly infected cases. We have $I(t) = \sum_{d=0}^{M} i(t,d)$. The removed cases will be counted separately for cure and death. Number of daily deaths and cured cases on day t is denoted by $\dot{D}_t$ and $\dot{C}_t$. The number of cumulative deaths on day t is $D(t) := D(t-1) + \dot{D}_t$ and cumulative cured case on day t is $C(t) := C(t-1) + \dot{C}_t$. The removed cases are the sum of cured and dead cases: $R(t) = C(t) + D(t)$. For the discrete time model, we define the conditional cure and mortality rate for infectious cases who have been infected for $x = d$ days by

$$c_d = \frac{F_1(d) - F_1(d-1)}{\bar{F}(d-1)} \qquad \text{and} \qquad m_d = \frac{F_2(d) - F_2(d-1)}{\bar{F}(d-1)} \tag{S10}$$

which serves as the discrete time versions of $h_1(x)$ and $h_2(x)$. Similarly, define the discrete time pseudo-density functions $f_1(x)$ and $f_2(x)$ by

$$f_c(d) = F_1(d) - F_1(d-1) \qquad \text{and} \qquad f_m(d) = F_2(d) - F_2(d-1). \tag{S11}$$

The discrete time contact rate $\beta(t)$ is defined as $\beta_t = B(t) - B(t-1)$ where $B(t) = \int_0^t \beta(s)ds$.

On day $t-1$, we have $N = S(t-1) + I(t-1) + D(t-1) + C(t-1)$. Moving to day t, the $I(t-1)$ cases transmit disease to the susceptible cases with rate β_t. Consequently, number of newly infected cases is

$$S(t-1) - S(t) = i(t,0) = \beta_t \frac{I(t-1)S(t-1)}{N}.$$

There are three possible outcomes for the $I(t-1)$ infectious cases on day t: cured, dead, or infectious (status quo), with probabilities depending on infectious duration. This yields the following decomposition. First observe that $I(t-1) = \sum_{d=0}^{M-1} i(t-1,d)$. For $i(t-1,d)$, the probability of cure is c_d, the probability of death is m_d and the probability of remaining infectious is $1 - c_d - m_d$. The infectious cases, $i(t-1,d) \times (1 - c_d - m_d)$, will be counted as $i(t, d+1)$ on day t because their infectious duration increases one day, i.e. $i(t,d+1) = i(t-1,d)\big(1 - c_d - m_d\big)$. The cured cases, $i(t-1,d)c_d$, and dead cases, $i(t-1,d)m_d$, are counted towards daily cured and dead cases on day t. That is,

$$\dot{C}_t = \sum_{d=0}^{M-1} i(t-1,d)c_d, \quad \text{and} \quad \dot{D}_t = \sum_{d=0}^{M-1} i(t-1,d)m_d.$$

In total, we have

$$
\begin{aligned}
& S(t)+I(t)+C(t)+D(t) \\
= \;& \big[S(t-1)-i(t,0)\big]+\Big[\sum_{d=0}^{M} i(t,d)\Big]+\big[C(t-1)+\dot{C}_t\big]+\big[D(t-1)+\dot{D}_t\big] \\
= \;& S(t-1)+\Big[\sum_{d=1}^{M} i(t,d)\Big]+\Big[C(t-1)+\sum_{d=0}^{M-1} i(t-1,d)c_d\Big] \\
& \quad +\Big[D(t-1)+\sum_{d=0}^{M-1} i(t-1,d)m_d\Big] \\
= \;& S(t-1)+C(t-1)+D(t-1)+\sum_{d=0}^{M-1}\Big[i(t-1,d)\big(1-c_d-m_d\big)\Big] \\
& +\sum_{d=0}^{M-1} i(t-1,d)c_d+\sum_{d=0}^{M-1} i(t-1,d)m_d \\
= \;& S(t-1)+C(t-1)+D(t-1)+\sum_{d=0}^{M-1} i(t-1,d) \\
= \;& S(t-1)+C(t-1)+D(t-1)+I(t-1)=N.
\end{aligned}
$$

To check that $dS/dt+dI/dt+dR/dt=0$ holds,

$$
\begin{aligned}
& \Big[S(t)-S(t-1)\Big]+\Big[I(t)-I(t-1)\Big]+\Big[\dot{C}_t+\dot{D}_t\Big] \\
= \;& \big[-i(t,0)\big]+\Big[i(t,0)+\sum_{d=0}^{M-1} i(t-1,d)\big(1-c_d-m_d\big)-\sum_{d=0}^{M-1} i(t-1,d)\Big] \\
& +\Big[\sum_{d=0}^{M-1} i(t-1,d)c_d+\sum_{d=0}^{M-1} i(t-1,d)m_d\Big] \\
= \;& 0.
\end{aligned}
$$

Algorithm 1 describes the discrete time algorithm for calculating $\{S(t), I(t), R(t), D(t), C(t) : t=1,\ldots,T_{\max}\}$ where $T_{\max}$ is maximum number of days under study.

Algorithm 1 Discrete Time Algorithm for SICD Model

```
Inputs:
    {N, D(0), C(0), {β_t}_1^{T_max}, {i(0,d)}_1^M, {c_d}_1^M, {m_d}_1^M}
Initialize:
    I(0) = Σ_{d=0}^{M} i(0,d), R(0) = D(0) + C(0), S(0) = N − I(0) − R(0)
for t = 1 to T_max do
    i(t,0) = β_t I(t−1) S(t−1)/N
    Ḋ_t = Σ_{v=1}^{M−1} i(t−1,v) m_v and Ċ_t = Σ_{v=1}^{M−1} i(t−1,v) c_v
    for v = 0 to M − 1 do
        i(t,v+1) = i(t−1,v)(1 − c_v − m_v)
    end for
    I(t) = Σ_{v=0}^{M} i(t,v), D(t) = D(0) + Σ_{s=1}^{t} Ḋ_s and C(t) = C(0) + Σ_{s=1}^{t} Ċ_s
    R(t) = D(t) + C(t) and S(t) = N − R(t) − I(t)
end for
return {S(t), I(t), i(t,d), R(t), D(t), C(t), Ċ(t), Ḋ(t)}_1^{T_max}
```

S2 Specification of c_d and m_d

Recall from Corollary 1 that the classical SIR model assumes an exponential distribution for X and constant hazards. Although the exponential distribution is widely used in survival analysis [41], in these applications the initial time is often recorded as the time point of hospitalization or diagnosis for a specific stage of disease development [42, 43], rather than the beginning of disease development. However the hazard rate at the beginning of disease development such as COVID-19 is often very low with increasing values later in time which makes a constant hazard assumption inappropriate for such settings.

S2.1 Lognormal distribution

Unlike the exponential distribution, the lognormal distribution [44, 45] can accomodate hazards that can first increase and then decrease over time [46]. Recall identity [S7] provides a method for specifying the CIF in terms of the hazard function of our chosing by specifying a random variable T_j with the target hazard. We use the lognormal distribution which corresponds to T_j that is assumed normally distributed under a log base-e transformation,

$$\ln T_j \sim \mathrm{N}(\mu_j, \sigma_j^2). \tag{S12}$$

Let $\phi_{\mu,\sigma}$ and $\Phi_{\mu,\sigma}$ denote the density and CDF for a $\mathrm{N}(\mu, \sigma^2)$ random variable. By [S7] we have

$$\begin{aligned}
F_1(x) &= \int_0^x \mathbb{P}\{T_2 \geq s\}\, d\mathbb{P}\{T_1 \leq s\} \\
&= \int_0^x \mathbb{P}\{\ln T_2 \geq \ln s\}\, d\mathbb{P}\{T_1 \leq s\} \\
&= \int_0^x [1 - \Phi_{\mu_2,\sigma_2}(\ln s)]\, d\mathbb{P}\{T_1 \leq s\} \\
&= \int_0^x d\mathbb{P}\{T_1 \leq s\} - \int_0^x \Phi_{\mu_2,\sigma_2}(\ln s)\, d\mathbb{P}\{T_1 \leq s\} \\
&= \mathbb{P}\{\ln T_1 \leq \ln x\} - \int_0^x \Phi_{\mu_2,\sigma_2}(\ln s)\, d\mathbb{P}\{\ln T_1 \leq \ln s\} \\
&= \Phi_{\mu_1,\sigma_1}(\ln x) - \int_0^x \Phi_{\mu_2,\sigma_2}(\ln s)\, \frac{1}{s}\, \phi_{\mu_1,\sigma_1}(\ln s)\, ds.
\end{aligned}$$

Similarly, we have $F_2(x) = \Phi_{\mu_2,\sigma_2}(\ln x) - \int_0^x \Phi_{\mu_1,\sigma_1}(\ln s)\, \frac{1}{s}\, \phi_{\mu_2,\sigma_2}(\ln s)\, ds$. Both F_1 and F_2 can be rapidly computed using standard software.

S2.2 Bimodal lognormal distribution

In a second approach we use a bimodal lognormal distribution [47]. This is done in consideration of two possibilities: (1) a subpopulation of infectious cases that are able to recover in a shorter period with low mortality rate; and (2) a subpopulation of infectious cases whose medical situation deteriorates requiring hospitalization care such that after treatment they are either cured or have relatively high mortality rate. Subpopulation (1) can reflect asymptomatic carriers [48–50] and/or carriers involving mutations [51, 52] and/or carriers of viruses with specific lineage [53, 54]. In order to model the lognormal bimodal distribution, T_j is assumed to satisfy

$$\ln T_j \sim p\mathrm{N}(\mu_{j1}, \sigma_{j1}^2) + (1-p)\mathrm{N}(\mu_{j2}, \sigma_{j2}^2). \tag{S13}$$

By [S7] we have

$$
\begin{aligned}
&F_1(x)\\
&\quad= \int_0^x \mathbb{P}\{T_2 \geq s\}\, d\mathbb{P}\{T_1 \leq s\}\\
&\quad= \int_0^x \mathbb{P}\{\ln T_2 \geq \ln s\}\, d\mathbb{P}\{T_1 \leq s\}\\
&\quad= \int_0^x \Big[1 - p\Phi_{\mu_{21},\sigma_{21}}(\ln s) - (1-p)\Phi_{\mu_{22},\sigma_{22}}(\ln s)\Big] d\mathbb{P}\{T_1 \leq s\}\\
&\quad= \int_0^x d\mathbb{P}\{\ln T_1 \leq \ln s\} - \int_0^x \Phi_{\mu_{22},\sigma_{22}}(\ln s)\, d\mathbb{P}\{\ln T_1 \leq \ln s\}\\
&\qquad +p \int_0^x \Big[\Phi_{\mu_{22},\sigma_{22}}(\ln s) - \Phi_{\mu_{21},\sigma_{21}}(\ln s)\Big] d\mathbb{P}\{\ln T_1 \leq \ln s\}\\
&\quad= p\, \Phi_{\mu_{11},\sigma_{11}}(\ln x) + (1-p)\Phi_{\mu_{12},\sigma_{12}}(\ln x)\\
&\qquad - \int_0^x \Phi_{\mu_{22},\sigma_{22}}(\ln s)\, \frac{1}{s} \Big[p\phi_{\mu_{11},\sigma_{11}}(\ln s) + (1-p)\phi_{\mu_{12},\sigma_{12}}(\ln s)\Big]\, ds\\
&\qquad +p \int_0^x \Big[\Phi_{\mu_{22},\sigma_{22}}(\ln x) - \Phi_{\mu_{21},\sigma_{21}}(\ln x)\Big]\, \frac{1}{s} \Big[p\phi_{\mu_{11},\sigma_{11}}(\ln s) + (1-p)\phi_{\mu_{12},\sigma_{12}}(\ln s)\Big]\, ds.
\end{aligned}
$$

Similarly, we have

$$
\begin{aligned}
&F_2(x)\\
&\quad= p\, \Phi_{\mu_{21},\sigma_{21}}(\ln x) + (1-p)\Phi_{\mu_{22},\sigma_{22}}(\ln x)\\
&\qquad - \int_0^x \Phi_{\mu_{12},\sigma_{12}}(\ln x)\, \frac{1}{s} \Big[p\phi_{\mu_{21},\sigma_{21}}(\ln x) + (1-p)\phi_{\mu_{22},\sigma_{22}}(\ln s)\Big]\, ds\\
&\qquad +p \int_0^x \Big[\Phi_{\mu_{12},\sigma_{12}}(\ln s) - \Phi_{\mu_{11},\sigma_{11}}(\ln s)\Big]\, \frac{1}{s} \Big[p\phi_{\mu_{21},\sigma_{21}}(\ln s) + (1-p)\phi_{\mu_{22},\sigma_{22}}(\ln s)\Big]\, ds.
\end{aligned}
$$

When $p = 1$, the above equations simplify to the unimodal case considered previously.

S2.3 Comparison of three survival models

We investigate daily infected and deaths calculated under three survival model scenarios:

1. *Exponential model (Scenario I).* In this scenario, we use the classical SIR model of Corollary 1 with $\lambda_1 = 0.032$, $\lambda_2 = 2.93 \times 10^{-3}$ and $\gamma = \lambda_1 + \lambda_2 = 0.034$.

2. *Lognormal model (Scenario II).* We use a lognormal as in [S12] with $\mu_1 = 3.506$, $\sigma_1 = 0.51$, $\mu_2 = 3.8$ and $\sigma_2 = 0.91$;

3. *Bimodal lognormal model (Scenario III).* We use a bimodal lognormal distribution as in [S13] with $p = 0.3$, $\mu_{11} = 1.908$, $\sigma_{11} = 0.4$, $\mu_{12} = 3.968$, $\sigma_{12} = 0.5$, $\mu_{21} = \mu_{22} = 3.8$ and $\sigma_{21} = \sigma_{22} = 0.91$.

For comparison, we adjust m_d and c_d for scenarios II and III so their mortality rates, M_{death}, and mean infectious periods, $\bar{X}$, are the same as those in scenario I. The mortality rate was 8.5% and mean infectious time was $\bar{X} = 29$ days. For scenarios II and III, we first calculate $F_1(x)$ and $F_2(x)$ and then adjust them by

$$
F_1(x) \leftarrow \frac{F_1(x) \times (1 - M_{\text{death}})}{F_1(T_{\max})} \text{ and } F_2(x) \leftarrow \frac{F_2(x) \times M_{\text{death}}}{F_2(T_{\max})}, \tag{S14}
$$

where $T_{\max}$ was set to 400. For the three scenarios, $F_1(x)$, $F_2(x)$ and $\bar{F}(x)$ are displayed in Fig S1. Discrete time values for f_c and f_m using [S11] are given in Fig S2A,B and discrete time values $\{c_d, m_d\}$ using equation [S10] are shown in Fig S2C,D. In the next section, we compare how the three survival models perform in modeling U.S. COVID-19 data.

S3 SICD model applied to pre-vaccination data

The three survival models were fit to COVID-19 New York Times data using Algorithm 1. Population size was set to N = 325,222,000 equal to the sum of populations for the states and regions reported by the New York Times. Values were intialized using using $i(0,0) = 1$, and $D(0) = C(0) = i(0,1) = i(0,2) = \cdots = i(0,M) = 0$. Values for $\{c_d, m_d\}_1^M$ were initialized according to the survival model used.

S3.1 Constant contact rate

As a preliminary step to understand the behavior of models we started with a simplified analysis that assumed a constant contact rate $\beta(t) = \beta_t = \beta = 0.2$. The mortality rate and mean infectious times were set to previous values $M_{\text{death}} = 8.5\%$ and $\bar{X} = 29$. Because all three models have the same infectious period, they have the same basic reproduction number $R_0 = \beta\bar{X} = 0.2 \times 29 = 5.8$. Results are shown in Figs. S3 and S4. In all three scenarios, nearly the entire population is infected within 300 days with 8.5% of infected dead. However, the outbreak ends faster under II than scenarios I and III (Fig S4). Maximum number of daily infected cases peaks differently among the three scenarios. Due to a constant contact rate, infected cases Fig S4D are mainly driven by the distribution of cure time (specified in Fig S2A,C). Within the first 20 days, scenario II assumes the lowest cure rate, therefore its peak of daily infectious cases is the highest among all scenarios. Daily deaths displayed as percentage of population are given in Fig S4B. The dynamics of daily death are driven by the survival specifications of death in Fig S2B,D. Because scenario II has the highest peak, this leads to the highest peak for daily death. Scenario I is the smallest, therefore it has the lowest peak in Fig S4B. Fig S4C displays aCDR, defined as cumulative deaths, $D(t)$, divided by cumulative infected cases, $D(t) + I(t) + C(t) = N - S(t)$. Values of aCDR should be very low at onset of disease due to few individual being cured or dying immediately after infection. Scenario I (exponential) does not conform to this and is therefore unrealistic.

S3.2 Data-driven time varying contact rate

A constant β_t is too simplistic for realistic modeling. This is because β_t is heavily influenced by lockdown measures and social distancing norms that change over time. Hence we apply the SICD model using a data driven β_t rate. Recall that β_t equals $[i(t,0)/I(t-1)] \times [N/S(t-1)]$, where $i(t,0)$ equals newly infected cases on day t. Because it is not easy to directly estimate this value, some form of approximation is necessary. The technique we use is to approximate $I(t)$ by the number infected cases reported within $\bar{X}$ days, since cases reported before $\bar{X}$ days are expected to be either cured or dead. Denote the number of daily new infected cases on record by I_t^{new}. We use the following data driven contact rate:

$$\beta_t = \frac{I_t^{\text{new}}}{\sum_{(s=t-\bar{X})}^{t} I_s^{\text{new}}}.$$

Values for β_t are displayed in Fig 2A. Fig S5 displays results from applying the discrete time algorithm using the data driven contact rate (all other parameter settings were the same as before). Scenarios I and III clearly underestimate reported daily infected cases, whereas scenario II estimates these values highly accurately. However all three methods perform poorly in estimating daily reported deaths: Scenarios I and III underestimate values, whereas II overestimates values.

The reason for the poor performance of I and III in estimating daily infections is that both assume a much faster recovery rate than II. Scenario II resolves this issue but as pointed out it still overestimates daily reported deaths. This suggested to us that a decrease in mortality rate must have occurred at some point in time, most likely after easing of lockdown measures. To test this, we set the mortality rate to $M_{\mathrm{death}} = 1.7\%$ for the second wave defined as the time period of May 9 through August 27. A value of 1.7% was also used for subsequent waves and data. One reason for selecting this particular time period was suggested by the behavior of the estimated contact rate of Fig 2A which displayed a wave like pattern over this time. To further investigate this, we estimated the basic reproduction number $R_0(t)$. Its values were estimated using discrete integral calculus using previous values for β_t, $f_m(d)$ and $f_c(d)$ for $t, d = 0, 1, \ldots, T_{\max}$ and where a mortality rate of 1.7% was used for data following first wave. Note that for recent dates, such as $R_0(t)$ on February 2nd in 2021, we have to provide β_s with $s \in [t, t + M]$ to estimate $R_0(t)$, for which we assume the existing wave pattern after May9th is repeated cyclically. Estimated $R_0(t)$ is given in Fig S6A. Notice that all three models have $R_0(t)$ values that begin roughly at the value of 1 at the start of second wave, and then increase and decrease and return to the value 1 in one full cycle over this time period. It is for this reason we refer to this period as the second wave. A similar pattern for $R_0(t)$ is observed for a third wave. We also note that values for $R_0(t)$ in Fig S6A are comparable to other studies. The R_0 for COVID-19 has been estimated previously at 1.3–6.5 with an approximate average of 3.3 [55, 56].

Fig 3F shows scenario II under the reduced mortality 1.7% for post first-wave data now fits observed values of aCDR, daily infected and deaths over the entire range of data. However, scenario I (Fig 3A) and scenario III (Fig S6C) still poorly approximate observed values. Thus there appears to be fundamental problems in applying these two models to COVID-19 data. Problems with these models are also evident from $R_0(t)$ of Fig S6A. The value of $R_0(t)$ is high in the first 70 days of the outbreak under all scenarios, which is realistic and expected, however $R_0(t)$ is much smaller for scenarios I and III compared with II. It is also worth noting that even though I and III have very similar $R_0(t)$ profiles, their estimated values for infected cases and deaths are quite different in Fig S6B. We mention that scenario III was considered under many different parameter settings. We found that even after trying different parameters, scenario III performed poorly unless the two modes for the cure event were very close. Thus we believe the assumption of bimodality is not justified for COVID-19. We conclude there is weak evidence supporting a bimodal distributed cure time where one subpopulation has quicker recovery time.

S4 Susceptible Infectious Vaccinated Cure Death Immune (SIVCDI) model for 2021 Vaccination Data

We extend the SICD model to include a vaccination compartment as follows:

$$
\begin{aligned}
\frac{dS^U}{dt} &= -\alpha(t)S^U - \frac{\beta^U(t)IS^U}{N} \\
\frac{dS^V}{dt} &= \alpha(t)S^U - \frac{\beta^V(t)IS^V}{N} - \eta(t)S^V \\
\frac{dI^U}{dt} &= \frac{\beta^U(t)IS^U}{N} - \gamma^U(t)I^U \\
\frac{dI^V}{dt} &= \frac{\beta^V(t)IS^V}{N} - \gamma^V(t)I^V \\
\frac{dR}{dt} &= \gamma^U(t)I^U + \gamma^V(t)I^V + \eta(t)S^V.
\end{aligned}
\tag{S15}
$$

Here the unvaccinated susceptible cases are denoted by S^U, the vaccinated susceptible cases are denoted by S^V, with $S = S^U + S^V$ as their sum, and likewise where unvaccinated infectious cases are denoted as I^U, vaccinated infectious are denoted as I^V, with $I = I^U + I^V$ as their

sum. The function $\alpha(t)$ is the vaccination rate at time t, β^U is the effective contact rate for unvaccinated cases at time t, equal to average number of contacts per person per time multiplied by probability of disease transmission between a unvaccinated susceptible and infectious case at time t, β^V is the contact rate between a vaccinated susceptible case and infectious case at time t, $\gamma^U(t)$ denotes the removal rate for unvaccinated infectious cases at time t, $\gamma^V(t)$ is the removal rate for the vaccinated infectious cases and $\eta(t)$ is the immune rate equal to percentage of vaccinated individuals who become immune to the disease. The sample size N is fixed and the above five equations can be reduced to four using $N = S^U + S^V + I + R$.

Because [S15] is an extension to include vaccination and immunity we refer to this as the Susceptible Infectious Vaccinated Cure Death Immune (SIVCDI) model. Previously for the SICD model, we used X as the continuous event time of an infected individual who either recovers from infection or dies due to infection. With the SIVCDI model, X becomes X_U and we add a new continuous variable X_V, defined as the event time for an infected vaccinated individual who either becomes cured or dies. Related to X_V is is another new variable E_V, defined as the event time for a vaccinated individual who becomes immune. We denote its density by $f_{E_V}(x)$ and its CDF by $F_{E_V}(x) = \mathbb{P}\{E_V \le x\}$. Additionally, define $E_V(t)$ to be the length of time a vaccinated individual has been vaccinated at time t. Denote its density by $f_{E_V(t)}(x)$ where $\int_0^\infty f_{E_V(t)}(x)dx = 1$. Using a similar argument as Theorem 1, we have

$$\eta(t) = \int_0^\infty \frac{f_{E_V}(x)}{1 - F_{E_V}(x)} f_{E_V(t)}(x)\,dx.$$

Similar to $\overline{X}$ of Theorem 3, define the mean immunity period as $\overline{E}_V = \int_0^\infty [1 - F_{E_V}(x)]dx$ which equals the average number of days a vaccinated individual who becomes immune requires to develop immunity. The parameter $\overline{E}_V$ can be influenced by brand of vaccine and timing of follow-up dose(s).

S4.1 Algorithm for a discrete time SIVCDI Model

Define $v(t, d') = S^V \int_{d'-1}^{d'} f_{E_V(t)}(x)\,dx$, $d' = 1, 2, \ldots, M$, as the number of vaccinated cases at time t who have been vaccinated for $x = d'$ days. As in section S1.3, M is a preset number chosen large enough so that $v(t, M+1) := S^V \int_M^\infty f_{E_V(t)}(x)dx$ can safely be assumed to equal zero. Let $v(t, 0)$ be the number of newly vaccinated cases. We have $S^V(t) = \sum_{d'=0}^{M} v(t, d')$.

For the discrete time model, we define the conditional immunity rate for vaccinated cases who have been vaccinated for $x = d'$ days by

$$e_{d'} = \frac{F_{E_V}(d') - F_{E_V}(d'-1)}{1 - F_{E_V}(d'-1)} \tag{S16}$$

The cumulative number of cases who are vaccinated immune at day t is

$$\dot{E}(t) = \sum_{d'=0}^{M-1} v(t-1, d') e_{d'}.$$

The number of vaccinated cases who become immune at day t is $E(t) = E(t-1) + \dot{E}(t)$. The removed cases will be counted separately for cure, death and additionally, vaccinated immunity: $R(t) = C(t) + D(t) + E(t)$.

Number of infectious cases at time t who have been infected for $x = d$ days, $i(t, d)$, described previously are now separated into $i^U(t, d)$ and $i^V(t, d)$ for unvaccinated and vaccinated infectious cases on day t with infected duration d, respectively. We also use separate conditional cure rates, c_d^U and c_d^V, and conditional death rates, m_d^U and m_d^V, for unvaccinated and vaccinated infectious cases. The update from $i(t-1, d)$ to $i(t, d+1)$ can be used for updating $i^U(t, d)$ and $i^V(t, d)$.

Similar to $i(t,d)$, values $v(t,d')$ are calculated iteratively. The number of newly vaccinated cases is

$$v(t,0) = \alpha_t S^U(t) = V_t^{\text{new}},$$

where α_t is the discrete time version of $\alpha(t)$. Because α_t is usually not available, we use newly vaccinated cases from public data to estimate this, this being denoted as V_t^{new}. For $v(t-1,d')$, the probability of immunity is $e_{d'}$ and the probability of remaining vaccinated is $1-e_{d'}$. The vaccinated cases, $v(t-1,d') \times (1-e_{d'})$, will be counted as $v(t,d'+1)$ on day t because their vaccinated duration increases one day, i.e. $v(t,d'+1) = v(t-1,d')\big(1-e_{d'}\big)$.

Algorithm 2 describes the discrete time algorithm for calculating

$$\{S^U(t), S^V(t), I(t), R(t), D(t), C(t), E(t) : t = 1, \ldots, T_{\max}\}$$

where as before $T_{\max}$ is maximum number of days under study.

Algorithm 2 Discrete Time Algorithm for the SIVCDI Model

Inputs:
$\{N,\ S^U(0),\ S^V(0),\ E(0),\ D(0),\ C(0),\ \{v(0,d)\}_1^M,\ \{i^U(0,d)\}_1^M,\ \{i^V(0,d)\}_1^M,$
$\{V_t^{\text{new}}\}_1^{T_{\max}},\ \{\beta_t^U\}_1^{T_{\max}},\ \{\beta_t^V\}_1^{T_{\max}},\ \{c_d^U\}_1^M,\ \{m_d^U\}_1^M,\ \{c_d^V\}_1^M,\ \{m_d^V\}_1^M,\ \{e_{d'}\}_1^M\}$

Initialize:
$I^U(0) = \sum_{d=0}^M i^U(0,d),\ I^V(0) = \sum_{d=0}^M i^V(0,d),$
$I(0) = I^U(0) + I^V(0),\ R(0) = D(0) + C(0) + E(0),$
$S^U(0) = N - S^V(0) - I(0) - R(0)$

for t = 1 to $T_{\max}$ **do**
  $v(t,0) = V_t^{\text{new}}$ and $\dot{E}_t = \sum_{d'=1}^{M-1} v(t-1,d')e_{d'}$
  $i^U(t,0) = \beta_t^U I(t-1)S^U(t-1)/N$ and $i^V(t,0) = \beta_t^V I(t-1)S^V(t-1)/N$
  $\dot{D}_t = \sum_{d=1}^{M-1} i^U(t-1,d)m_d^U + \sum_{d=1}^{M-1} i^V(t-1,d)m_d^V$
  $\dot{C}_t = \sum_{d=1}^{M-1} i^U(t-1,d)c_d^U + \sum_{d=1}^{M-1} i^V(t-1,d)c_d^V$
  for w = 0 to $M-1$ **do**
    $v(t,w+1) = v(t-1,w)\big(1-e_w\big)$
    $i^U(t,w+1) = i^U(t-1,w)\big(1-c_w^U-m_w^U\big)$
    $i^V(t,w+1) = i^V(t-1,w)\big(1-c_w^V-m_w^V\big)$
  end for
  $S^V(t) = \sum_{d'=0}^M v(t,d'),\ E(t) = E(0) + \sum_{s=1}^t \dot{E}_s$
  $I^U(t) = \sum_{d=0}^M i^U(t,d),\ I^V(t) = \sum_{d=0}^M i^V(t,d),\ I(t) = I^U(t) + I^V(t),$
  $D(t) = D(0) + \sum_{s=1}^t \dot{D}_s$ and $C(t) = C(0) + \sum_{s=1}^t \dot{C}_s$
  $R(t) = D(t) + C(t) + E(t)$ and $S^U(t) = N - S^V(t) - I(t) - R(t)$
end for
return $\{S^U(t),\ S^V(t),\ v(t,d'),\ I(t),\ i^U(t,d),\ i^V(t,d),\ R(t),\ D(t),\ C(t),\ E(t)\}_1^{T_{\max}}$

S4.2 Specification of β_t^U, β_t^V and $e_{d'}$

We use published vaccine studies to estimate β_t^U and β_t^V. Let $a\%$ and $b\%$ be percentages of infected case in the vaccine and control group, respectively. We can assume $\beta_t^V/\beta_t^U = a/b$. The contact rate β_t, is a weighted average of β_t^U and β_t^V,

$$\frac{S^U(t-1)}{S^U(t-1)+S^V(t-1)}\beta_t^U + \frac{S^V(t-1)}{S^U(t-1)+S^V(t-1)}\beta_t^V = \beta_t,$$

We calculate β_t as in the SICD model and by updating $S^U(t-1)$ and $S^V(t-1)$ iteratively we obtain data driven β_t^U and β_t^V. A recent vaccine cohort study [57] found an incidence rate of 4.7 vs 149.8 per 100,000 person-days for vaccinated to unvaccinated for symptomatic SARS-CoV-2. The study also reported an incidence rate of 11.3 vs 67.0 per 100,000 person-days for asymptomatic infection. Thus we obtain the ratio of percentages of infected cases for vaccinated group and control group as $a/b = (4.7 + 11.3)/(149.8 + 67)$.

For the immune rate $e_{d'}$, we assume an exponential function $F(x) = 1 - e^{-\zeta x}$. The mean expected time for exponential distribution is $\overline{E}_V = 1/\zeta$, so ζ is identifiable and estimatable. We assume the mean immunity period is $\overline{E}_V = 30$ days, thus yielding $e_{d'} = e^{-1/30} - 1 = 0.0339$. For the current data, our results were not particularly sensitive to $\overline{E}_V$. We obtained V_t^{new} from publicly available data [58]. As has been widely observed, mortality rate for vaccinated is near zero, thus we set the death rate for this group to $M_{\mathrm{death}} = 0.001\%$. Based on our what-if analysis we use a reduced cure time of 25 days. All other parameters including parameters for the unvaccinated infecious cases were set as before for the pre-vaccination analysis. The results are shown in Fig 6 and show a near perfect fit.

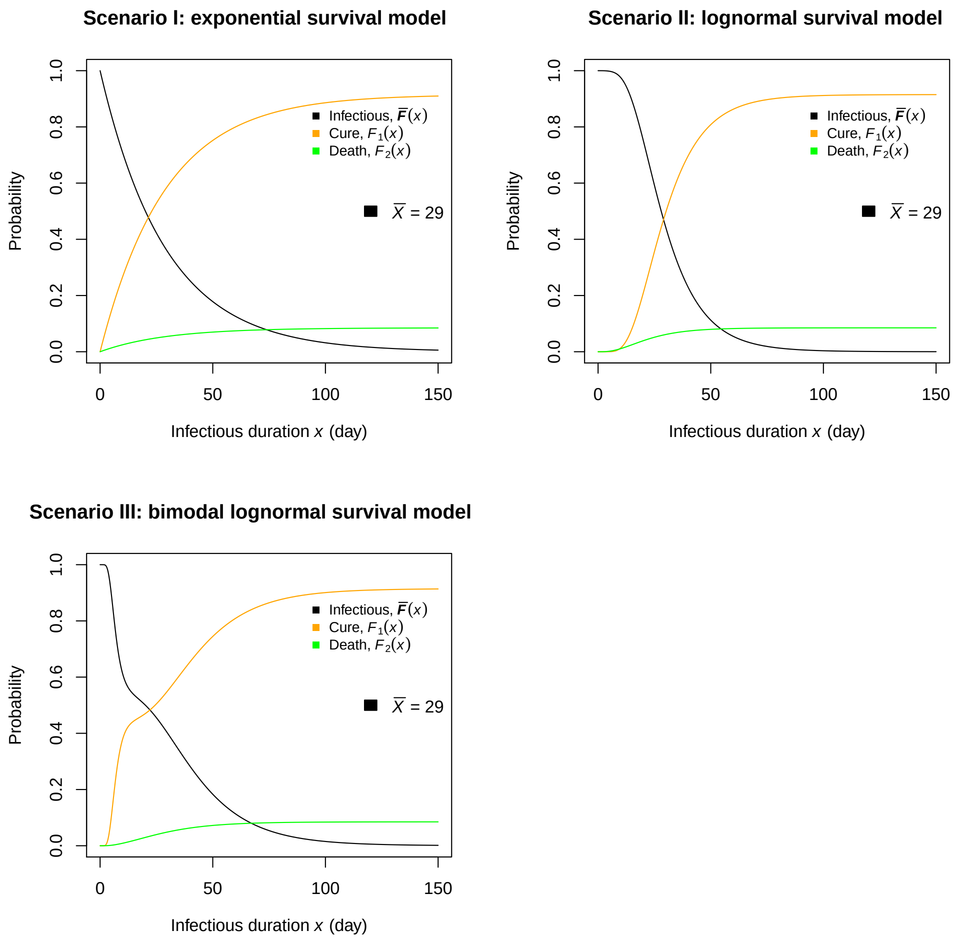

Fig S1. Comparison of three different survival models where all models have identical mean infectious period $\bar{X} = 29$ and mortality rate $M_{\text{death}} = 8.5\%$. Shown are CCDF $\bar{F}(t)$ (black), CIF for cure $F_1(t)$ (orange) and CIF for death $F_2(t)$ (green). Scenario I uses an exponential distribution, which is equivalent to the classical SIR model by Corollary 1. Scenario II uses a lognormal distribution. Scenario III uses a bimodal lognormal distribution.

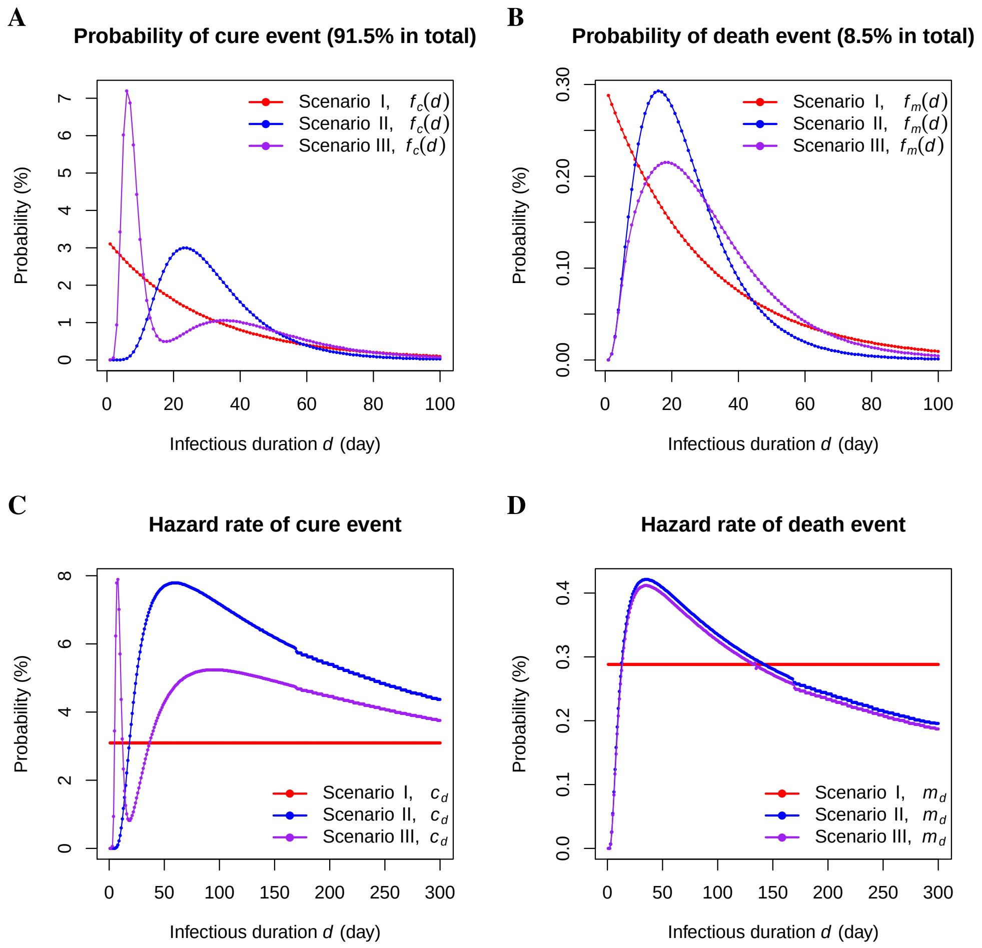

Fig S2. Discrete time survival values for scenario I (red), II (blue) and III (purple). (A) Discrete time pseudo-densities for cure. Most infections recover at the beginning in scenario I; around 15-40 days in scenario II; and either within 17 days, or around 25 to 55 days, in scenario III. (B) Discrete time pseudo-densities for death. Most deaths occur at the beginning in scenario I and around 15-40 days in scenarios II and III. (C) Discrete time hazard rates for cure. Scenario I has constant hazard whereas scenarios II and III assume hazards that initially increase and then decrease. Scenario III assumes a bimodal shape for the cure hazard. (D) Discrete time hazard rates for death.

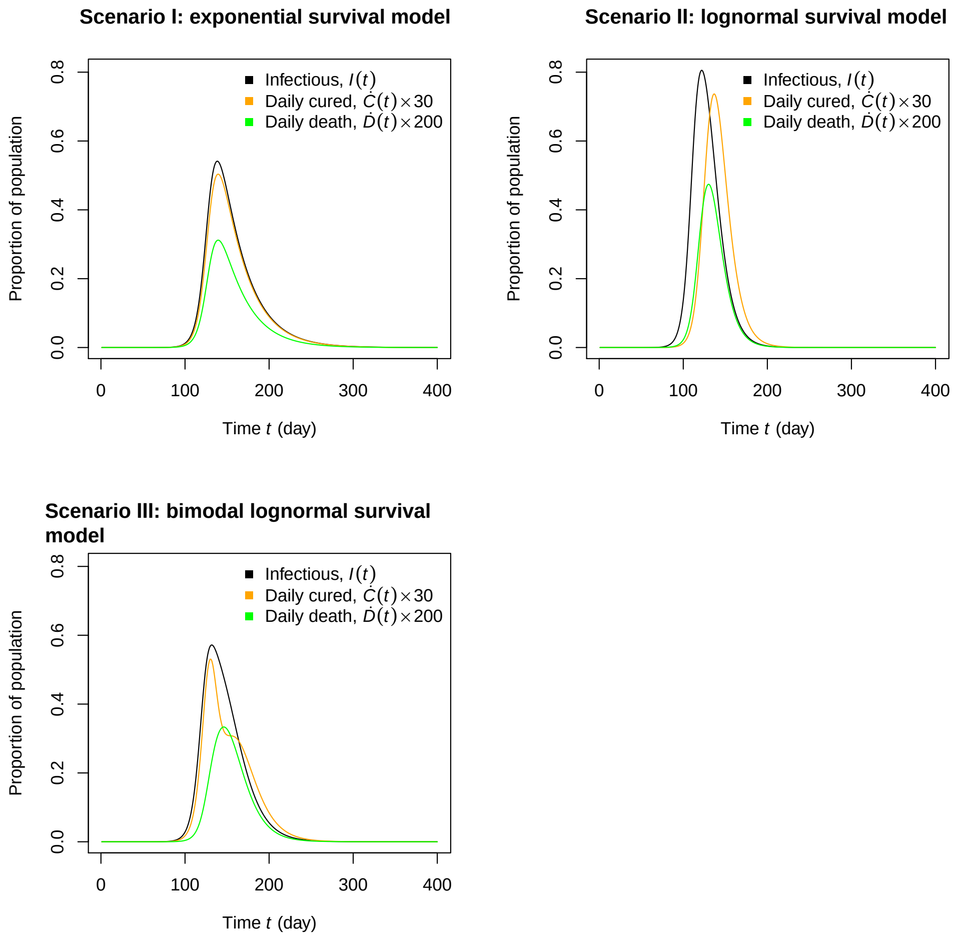

Fig S3. Discrete time SIR models assuming a constant contact rate $\beta(t) = 0.2$. Infectious cases $I(t)$ (black), daily cured cases $\dot{C}(t)$ (orange) and daily deaths $\dot{D}(t)$ (green) are displayed as percentage of total population. Daily cured and deaths being much smaller than $I(t)$ are multiplied by 30 and 200. In scenario I, all values have the same trend and peak at the same time. In scenario II, daily deaths peak after infectious cases, which is more realistic. In scenario III, deaths also peak after infectious cases, but daily cured has two waves due to the bimodal distribution assumption.

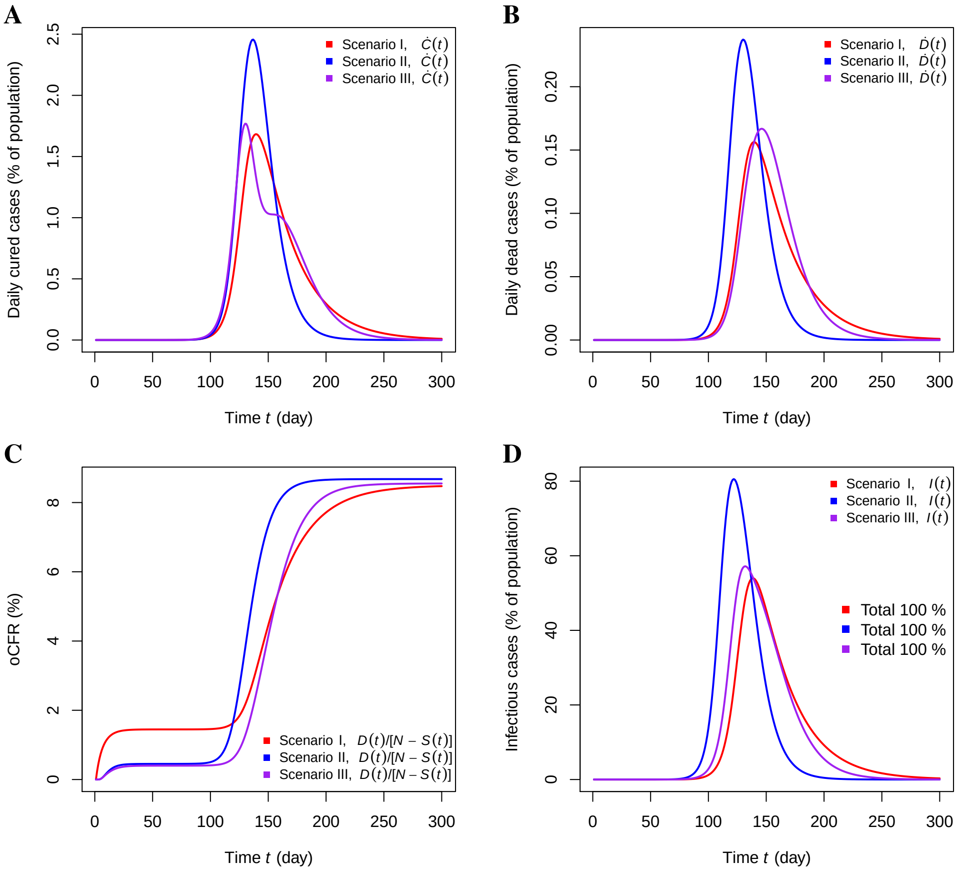

Fig S4. Comparison of discrete time SIR models assuming a constant contact rate $\beta(t) = 0.2$. (A) Daily cured. (B) Daily deaths. (C) aCDR. (D) Infectious cases as percentage of population, $I(t)/N$. Values of aCDR should be very low at onset of disease due to few cures and death occuring immediately after infection. Therefore, scenario I is unrealistic.

Scenario I: exponential survival model

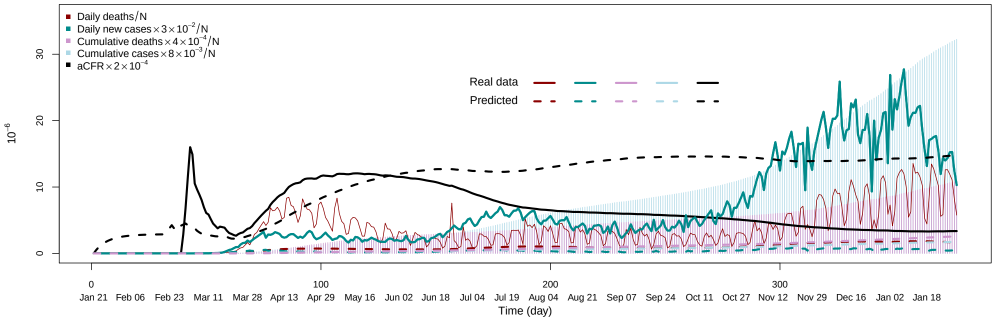

Scenario II: lognormal survival model

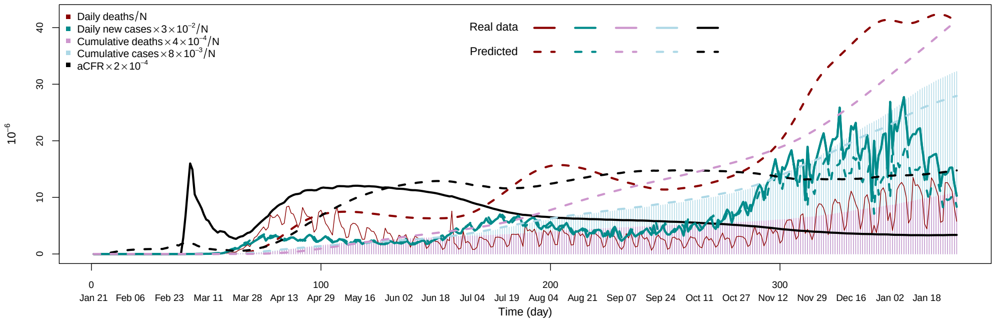

Scenario III: bimodal lognormal survival model

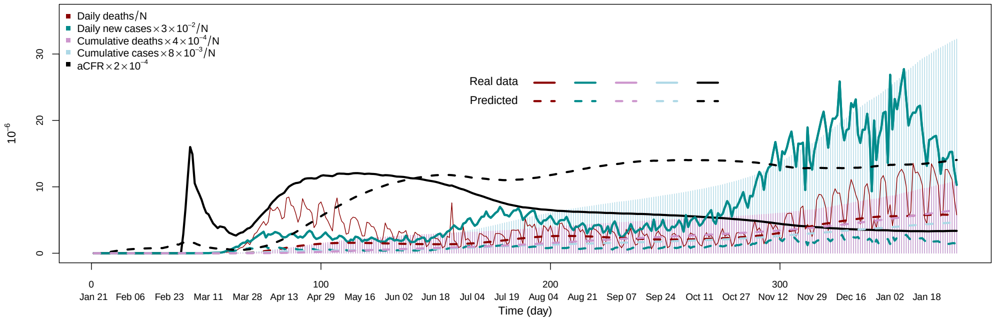

Fig S5. Analysis of COVID-19 pre-vaccination data assuming a constant mortality rate for first and subsequent waves. Scenario II is best at estimating daily new cases. However, aCDR and daily deaths are overestimated after first wave, thus suggesting a lower mortality for post-first wave data.

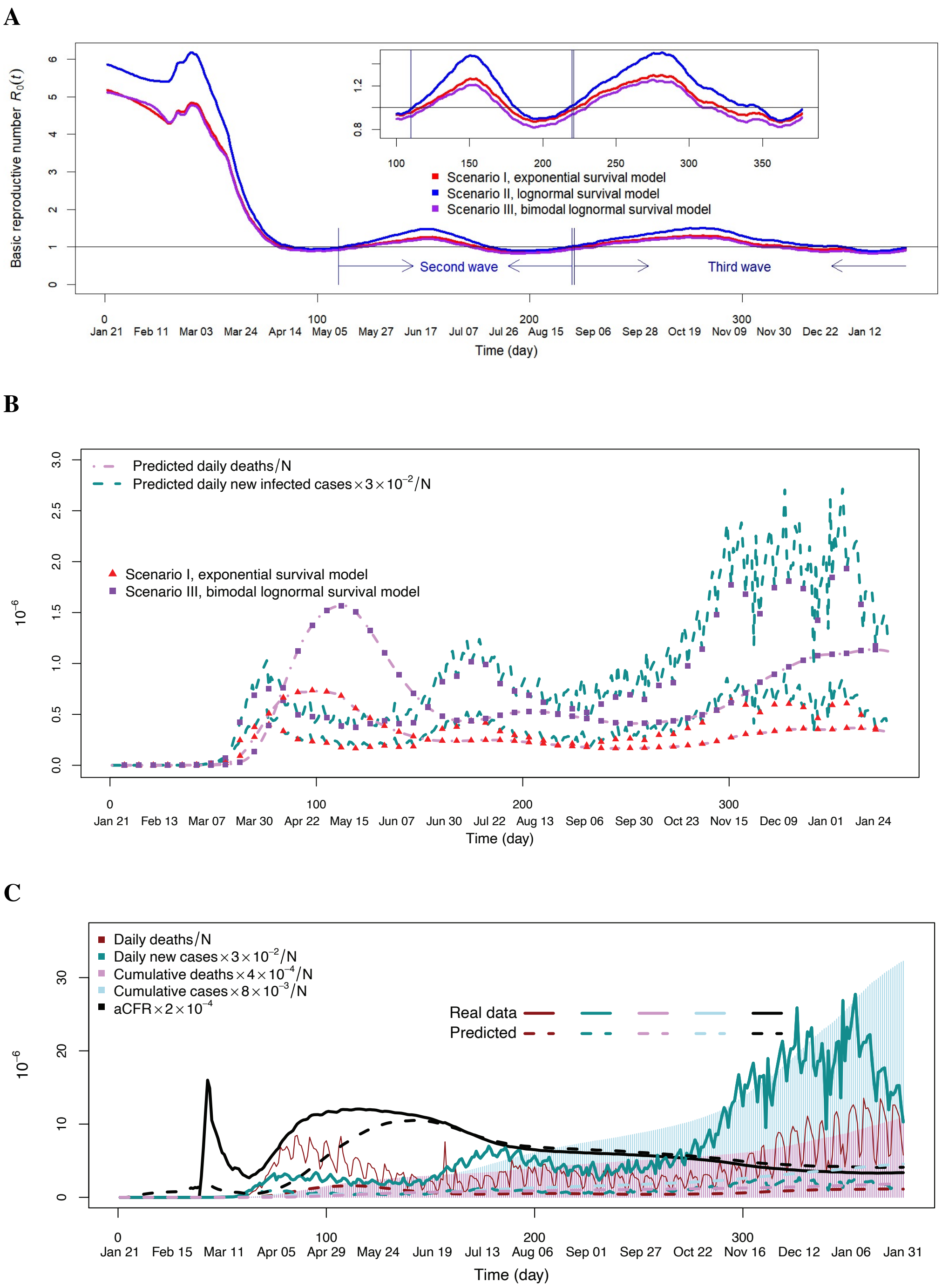

Fig S6. Analysis of COVID-19 pre-vaccination data assuming a lower mortality rate for second and subsequent waves. (A) Basic reproduction number $R_0(t)$; note its values are much smaller for Scenarios I and III than II. (B) Even though scenarios I and III have similar $R_0(t)$ profiles, estimated values for daily new infections and deaths are different. (C) Bimodal lognormal distribution continues to perform poorly even under assumption of lower mortality for post-first wave data.

References

32. Hethcote HW. The mathematics of infectious diseases. SIAM review. 2000;42(4):599–653.

33. Kermack WO, McKendrick AG. A contribution to the mathematical theory of epidemics. Proceedings of the Royal Society of London Series A, Containing Papers of a Mathematical and Physical Character. 1927;115(772):700–721.

34. Anderson RM, Anderson B, May RM. Infectious Diseases of Humans: Dynamics and Control. Oxford University Press; 1992.

35. Diekmann O, Heesterbeek J. Mathematical Epidemiology of Infectious Diseases: Model Building, Analysis, and Interpretation. John Wiley and Sons, Inc., New York; 2000.

36. Fraser C, Donnelly CA, Cauchemez S, Hanage WP, Van Kerkhove MD, Hollingsworth TD, et al. Pandemic potential of a strain of influenza A (H1N1): early findings. Science. 2009;324(5934):1557–1561.

37. Delamater PL, Street EJ, Leslie TF, Yang YT, Jacobsen KH. Complexity of the basic reproduction number (R0). Emerging Infectious Diseases. 2019;25(1):1.

38. Guerra FM, Bolotin S, Lim G, Heffernan J, Deeks SL, Li Y, et al. The basic reproduction number (R0) of measles: a systematic review. The Lancet Infectious Diseases. 2017;17(12):e420–e428.

39. Royston P, Parmar MK. Restricted mean survival time: an alternative to the hazard ratio for the design and analysis of randomized trials with a time-to-event outcome. BMC Medical Research Methodology. 2013;13(1):152.

40. Anastassopoulou C, Russo L, Tsakris A, Siettos C. Data-based analysis, modelling and forecasting of the COVID-19 outbreak. PLOS One. 2020;15(3):e0230405.

41. Glasser M. Exponential survival with covariance. Journal of the American Statistical Association. 1967;62(318):561–568.

42. Crowley J, Hu M. Covariance analysis of heart transplant survival data. Journal of the American Statistical Association. 1977;72(357):27–36.

43. Collett D. Modelling Survival Data in Medical Research. CRC press; 2015.

44. Chapman J, O'callaghan C, Hu N, Ding K, Yothers G, Catalano P, et al. Innovative estimation of survival using log-normal survival modelling on ACCENT database. British Journal of Cancer. 2013;108(4):784–790.

45. Royston P. The lognormal distribution as a model for survival time in cancer, with an emphasis on prognostic factors. Statistica Neerlandica. 2001;55(1):89–104.

46. Aragao GM, Corradini MG, Normand MD, Peleg M. Evaluation of the Weibull and log normal distribution functions as survival models of Escherichia coli under isothermal and non isothermal conditions. International Journal of Food Microbiology. 2007;119(3):243–257.

47. Fischer A, Abel A, Lepper M, Zitzelsberger A, Von Glasow A. Modeling bimodal electromigration failure distributions. Microelectronics Reliability. 2001;41(3):445–453.

48. Bai Y, Yao L, Wei T, Tian F, Jin DY, Chen L, et al. Presumed asymptomatic carrier transmission of COVID-19. JAMA. 2020;323(14):1406–1407.

49. Gandhi M, Yokoe DS, Havlir DV. Asymptomatic transmission, the Achilles heel of current strategies to control COVID-19; 2020.

50. Treibel TA, Manisty C, Burton M, McKnight Á, Lambourne J, Augusto JB, et al. COVID-19: PCR screening of asymptomatic health-care workers at London hospital. The Lancet. 2020;395(10237):1608–1610.

51. Grubaugh ND, Hanage WP, Rasmussen AL. Making sense of mutation: what D614G means for the COVID-19 pandemic remains unclear. Cell. 2020;.

52. Pachetti M, Marini B, Benedetti F, Giudici F, Mauro E, Storici P, et al. Emerging SARS-CoV-2 mutation hot spots include a novel RNA-dependent-RNA polymerase variant. Journal of Translational Medicine. 2020;18:1–9.

53. Tang X, Wu C, Li X, Song Y, Yao X, Wu X, et al. On the origin and continuing evolution of SARS-CoV-2. National Science Review. 2020;.

54. Koutsakos M, Kedzierska K. A race to determine what drives COVID-19 severity; 2020.

55. Sen-Crowe B, McKenney M, Elkbuli A. Social distancing during the COVID-19 pandemic: Staying home save lives. The American Journal of Emergency Medicine. 2020;.

56. Liu Y, Gayle AA, Wilder-Smith A, Rocklöv J. The reproductive number of COVID-19 is higher compared to SARS coronavirus. Journal of Travel Medicine. 2020;.

57. Angel Y, Spitzer A, Henig O, Saiag E, Sprecher E, Padova H, et al. Association Between Vaccination With BNT162b2 and Incidence of Symptomatic and Asymptomatic SARS-CoV-2 Infections Among Health Care Workers. JAMA. 2021;.

58. Hannah R, Esteban OO, Diana B, Edouard M, Joe H, Bobbie M, et al. Coronavirus Pandemic (COVID-19). Our World in Data. 2020;.
