# Supplementary material for: Cure and death play a role in understanding dynamics for COVID-19: Data-driven competing risk compartmental models, with and without vaccination
Source: PLoS One. 2021 Jul 15;16(7):e0254397. doi: 10.1371/journal.pone.0254397 (PMC8282006; doi:10.1371/journal.pone.0254397)
Supplement: S1 Fig — Shown are CCDF F¯(t) (black), CIF for cure F1(t) (orange) and CIF for death F2(t) (green). (A) Scenario I uses an exponential distribution, which is equivalent to the classical SIR model by Corollary 1 of Appendix. (B) Scenario II uses a lognormal distribution. (C) Scenario III uses a bimodal lognormal distribution. (PDF) [file pone.0254397.s004.pdf]

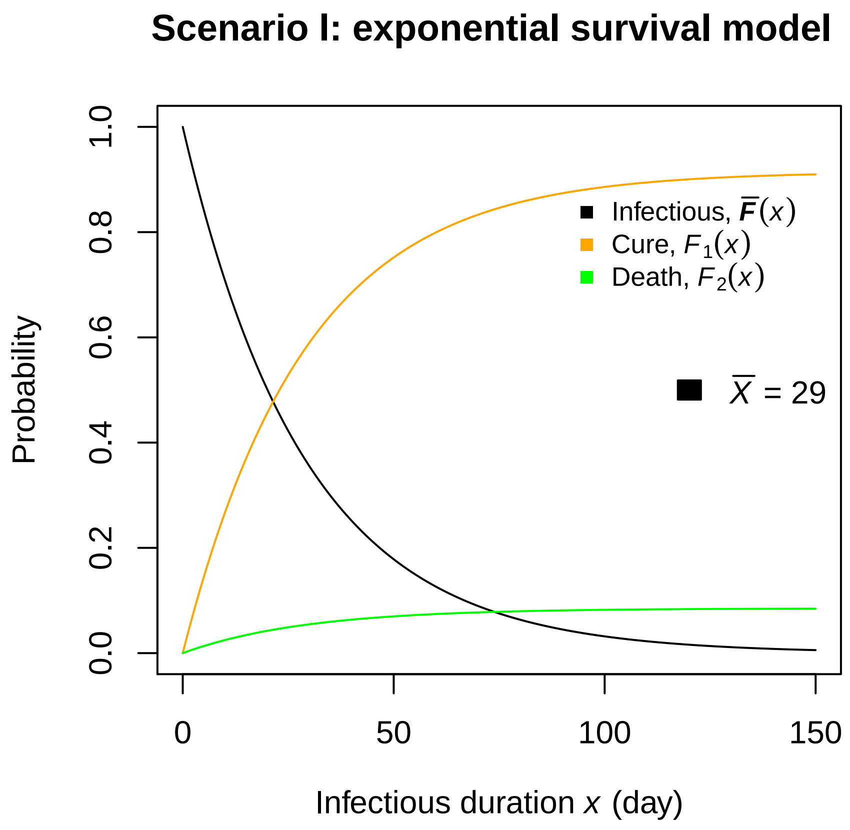

Scenario I: exponential survival model
Probability
1.0
0.8
0.6
0.4
0.2
0.0
0
50
100
150
Infectious duration x (day)
Infectious, $\bar{F}(x)$
Cure, $F_1(x)$
Death, $F_2(x)$
$\bar{X}$ = 29

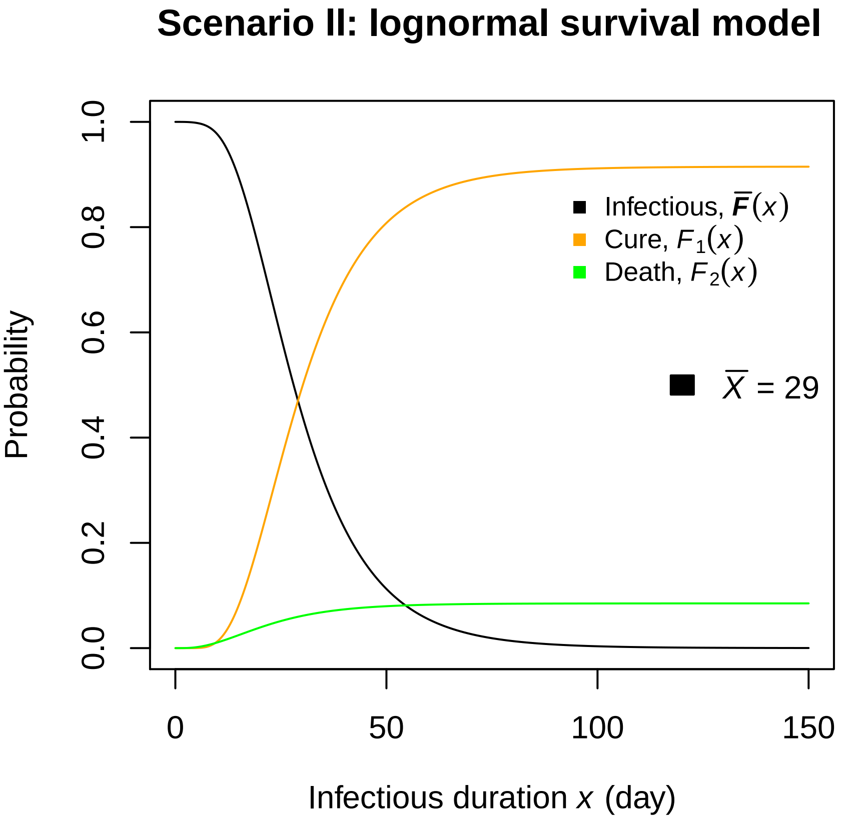

Scenario II: lognormal survival model
Infectious, $\bar{F}(x)$
Cure, $F_1(x)$
Death, $F_2(x)$
$\bar{X}$ = 29
Probability
0.0
0.2
0.4
0.6
0.8
1.0
0
50
100
150
Infectious duration x (day)

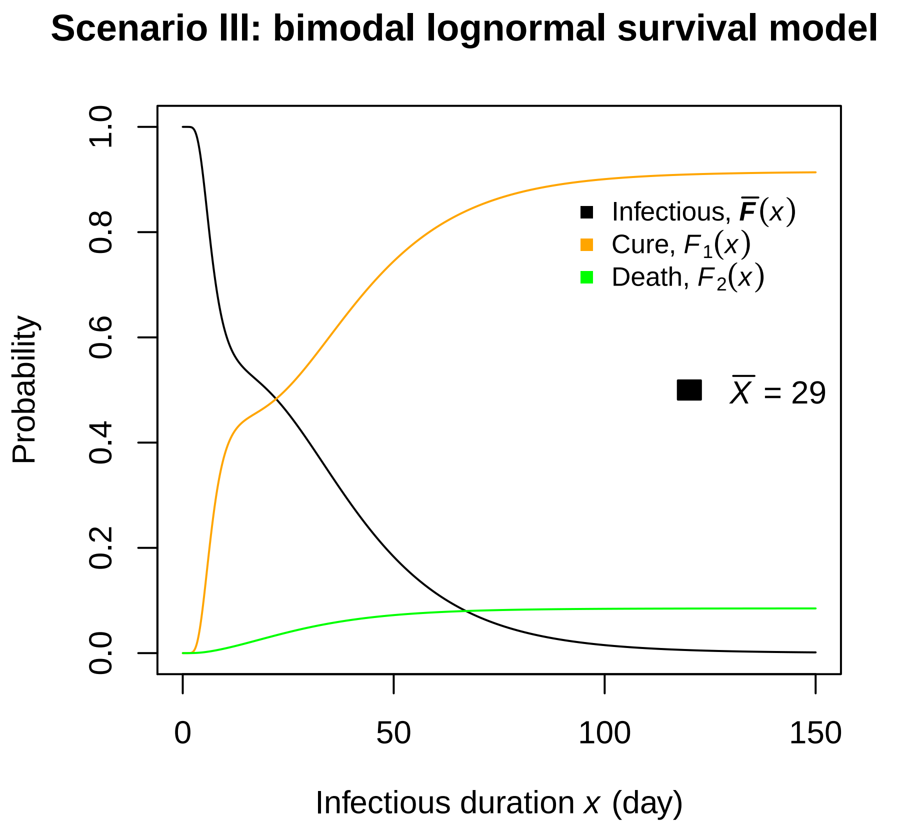
Scenario III: bimodal lognormal survival model
Infectious, $\bar{F}(x)$
Cure, $F_1(x)$
Death, $F_2(x)$
$\bar{X}$ = 29
Probability
1.0
0.8
0.6
0.4
0.2
0.0
0
50
100
150
Infectious duration x (day)
