# Supplementary material for: Cure and death play a role in understanding dynamics for COVID-19: Data-driven competing risk compartmental models, with and without vaccination
Source: PLoS One. 2021 Jul 15;16(7):e0254397. doi: 10.1371/journal.pone.0254397 (PMC8282006; doi:10.1371/journal.pone.0254397)
Supplement: S2 Fig — (A) Discrete time pseudo-densities for cure. Most infections recover at the beginning in scenario I; around 15-40 days in scenario II; and either within 17 days, or around 25 to 55 days, in scenario III. (B) Discrete time pseudo-densities for death. Most deaths occur at the beginning in scenario I and around 15-40 days in scenarios II and III. (C) Discrete time hazard rates for cure. Scenario I has constant hazard whereas scenarios II and III assume hazards that initially increase and then decrease. Scenario III assumes a bimodal shape for the cure hazard. (D) Discrete time hazard rates for death. (PDF) [file pone.0254397.s005.pdf]

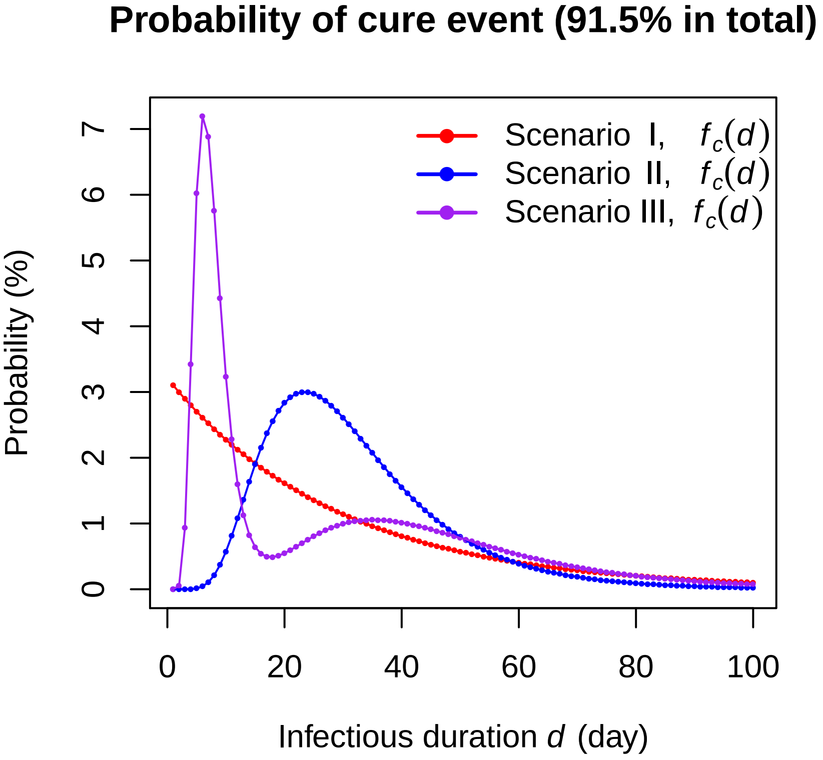

Probability of cure event (91.5% in total)
Scenario I, $f_c(d)$
Scenario II, $f_c(d)$
Scenario III, $f_c(d)$
Probability (%)
0
1
2
3
4
5
6
7
0
20
40
60
80
100
Infectious duration d (day)

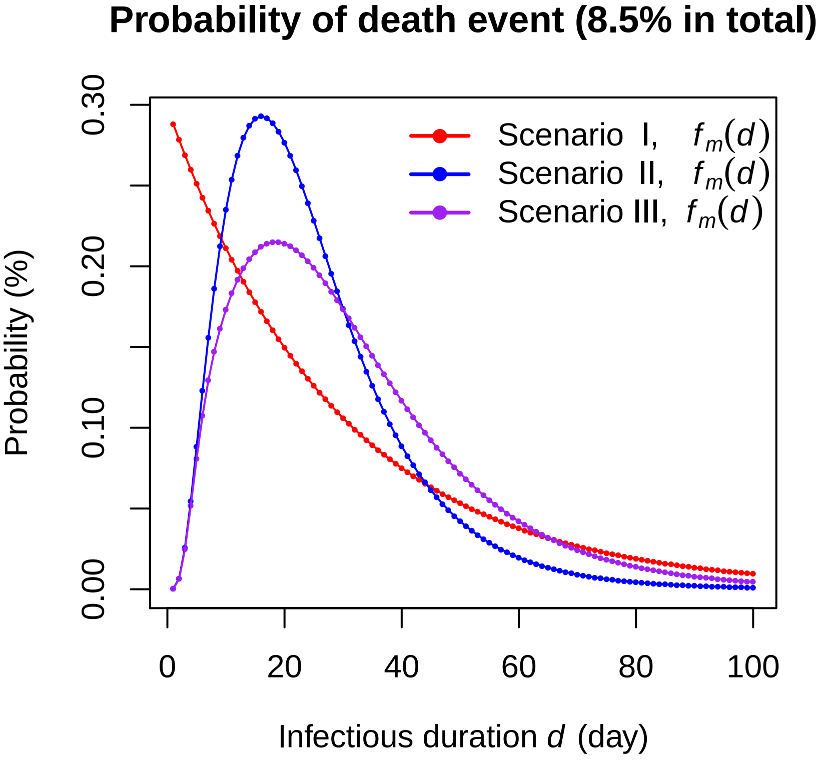

Probability of death event (8.5% in total)
Scenario I, $f_m(d)$
Scenario II, $f_m(d)$
Scenario III, $f_m(d)$
Probability (%)
0.00
0.10
0.20
0.30
0
20
40
60
80
100
Infectious duration d (day)

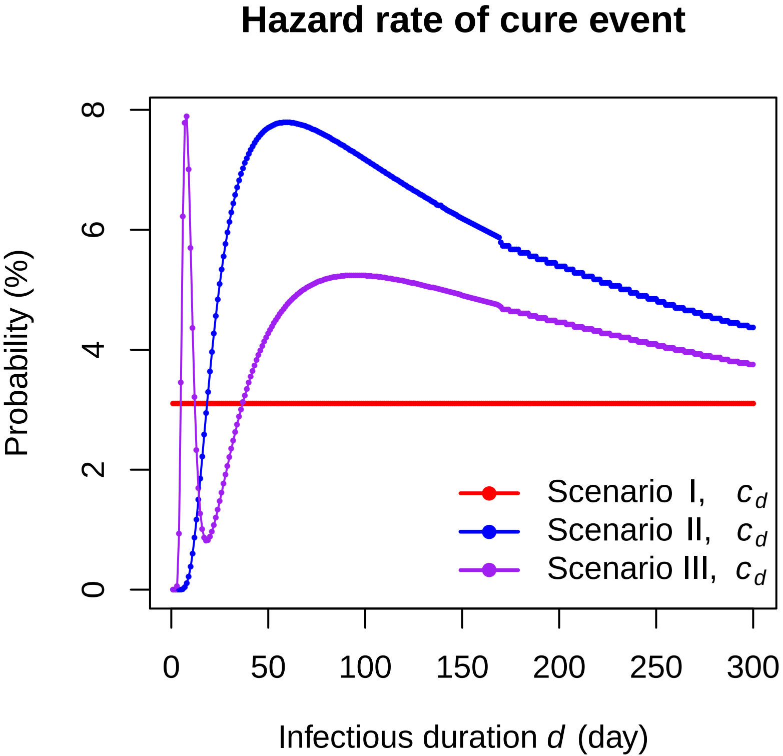

Hazard rate of cure event
Probability (%)
0
2
4
6
8
0
50
100
150
200
250
300
Infectious duration d (day)
Scenario I, c_d
Scenario II, c_d
Scenario III, c_d

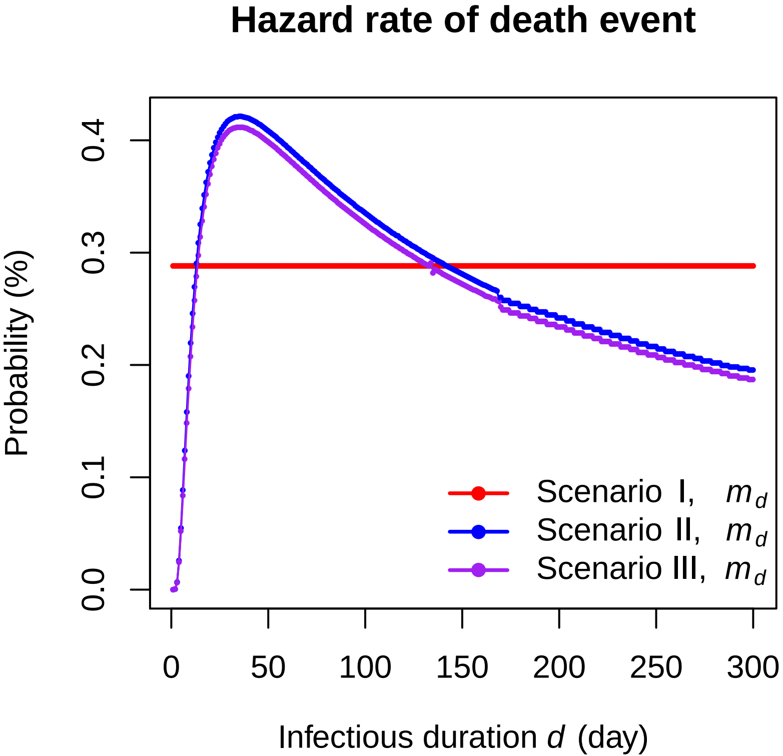

Hazard rate of death event
Probability (%)
0.0
0.1
0.2
0.3
0.4
0
50
100
150
200
250
300
Infectious duration d (day)
Scenario I, m_d
Scenario II, m_d
Scenario III, m_d
