# Supplementary material for: Cure and death play a role in understanding dynamics for COVID-19: Data-driven competing risk compartmental models, with and without vaccination
Source: PLoS One. 2021 Jul 15;16(7):e0254397. doi: 10.1371/journal.pone.0254397 (PMC8282006; doi:10.1371/journal.pone.0254397)
Supplement: S3 Fig — Infectious cases I(t) (black), daily cured cases C˙(t) (orange) and daily deaths D˙(t) (green) are displayed as percentage of total population. Daily cured and deaths being much smaller than I(t) are multiplied by 30 and 200. (A) In scenario I, all values have the same trend and peak at the same time. (B) In scenario II, daily deaths peak after infectious cases, which is more realistic. (C) In scenario III, deaths also peak after infectious cases, but daily cured has two waves due to the bimodal distribution assumption. (PDF) [file pone.0254397.s006.pdf]

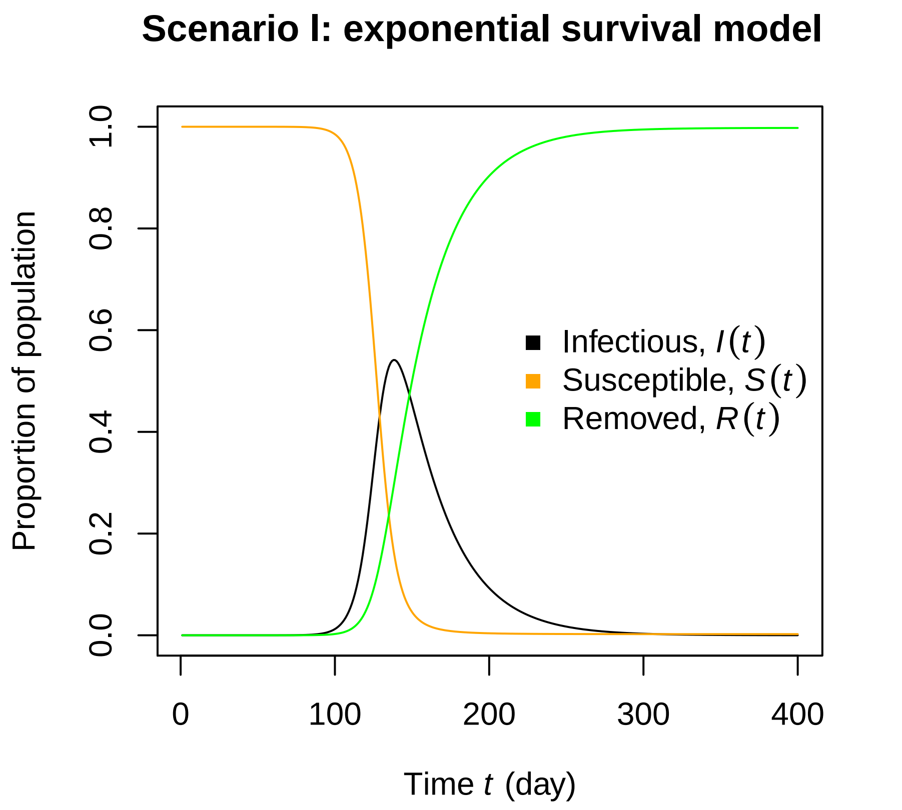

Scenario I: exponential survival model
Proportion of population
0.0
0.2
0.4
0.6
0.8
1.0
0
100
200
300
400
Time $t$ (day)
Infectious, $I(t)$
Susceptible, $S(t)$
Removed, $R(t)$

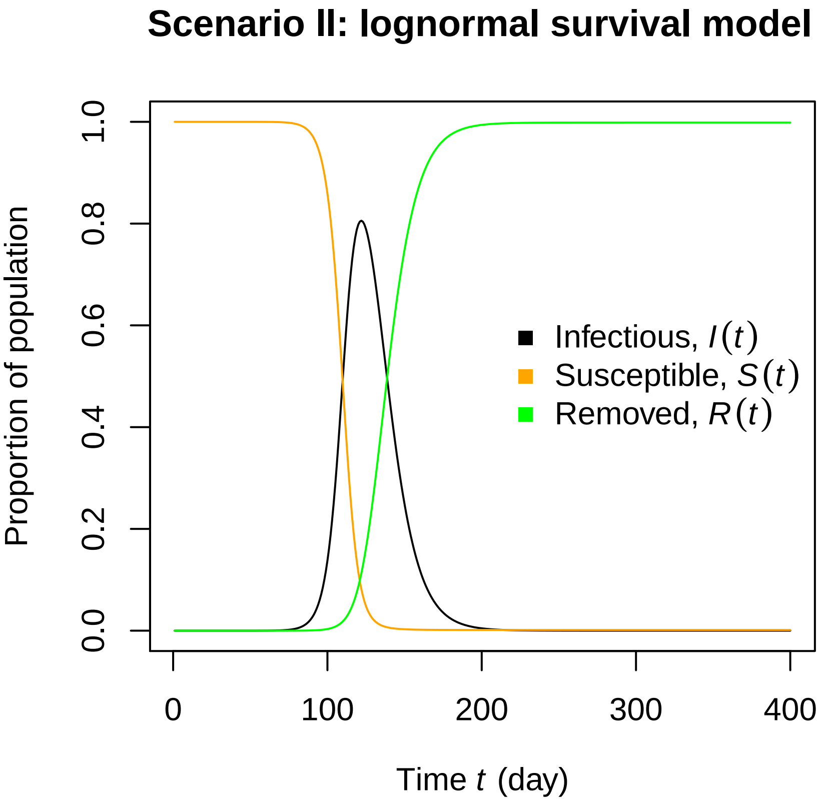

Scenario II: lognormal survival model
Proportion of population
0.0
0.2
0.4
0.6
0.8
1.0
0
100
200
300
400
Time t (day)
Infectious, I(t)
Susceptible, S(t)
Removed, R(t)

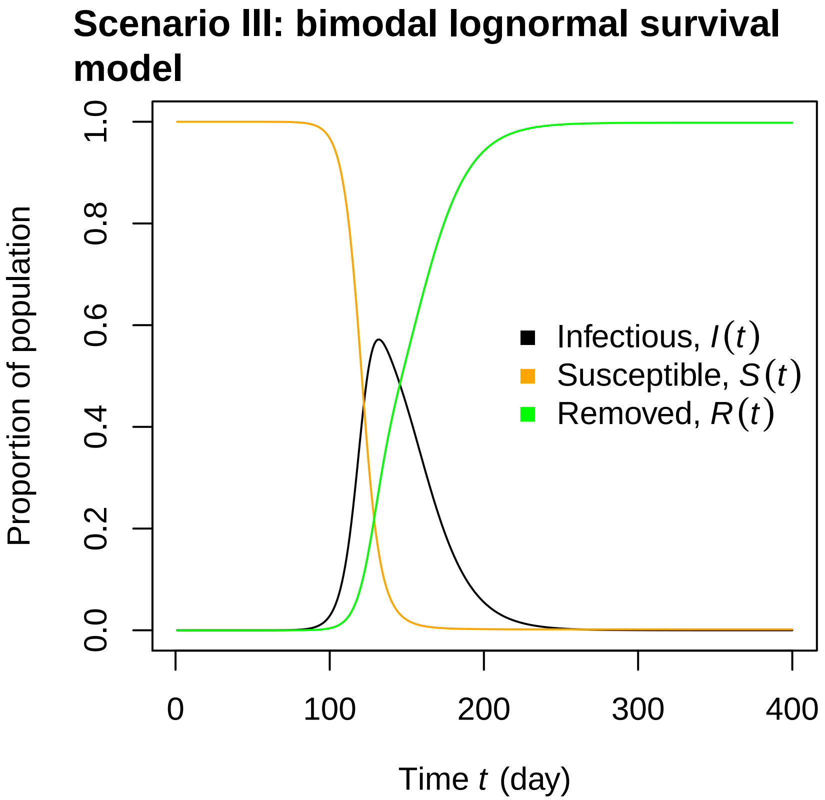
Scenario III: bimodal lognormal survival model
Proportion of population
0.0
0.2
0.4
0.6
0.8
1.0
0
100
200
300
400
Time $t$ (day)
Infectious, $I(t)$
Susceptible, $S(t)$
Removed, $R(t)$

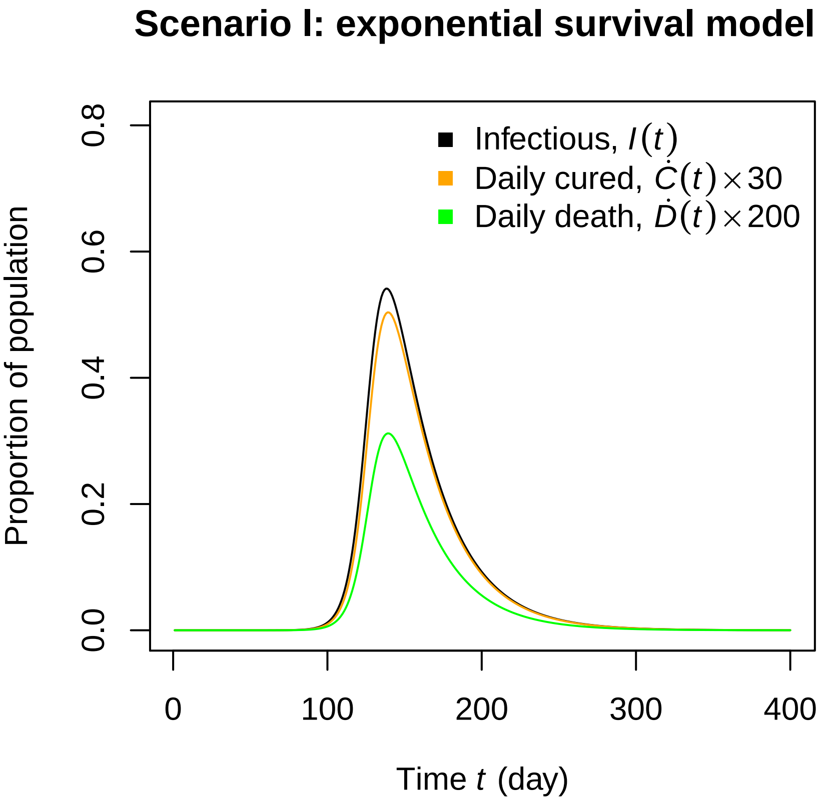

Scenario I: exponential survival model
Infectious, $I(t)$
Daily cured, $\dot{C}(t) \times 30$
Daily death, $\dot{D}(t) \times 200$
Proportion of population
0.0
0.2
0.4
0.6
0.8
0
100
200
300
400
Time $t$ (day)

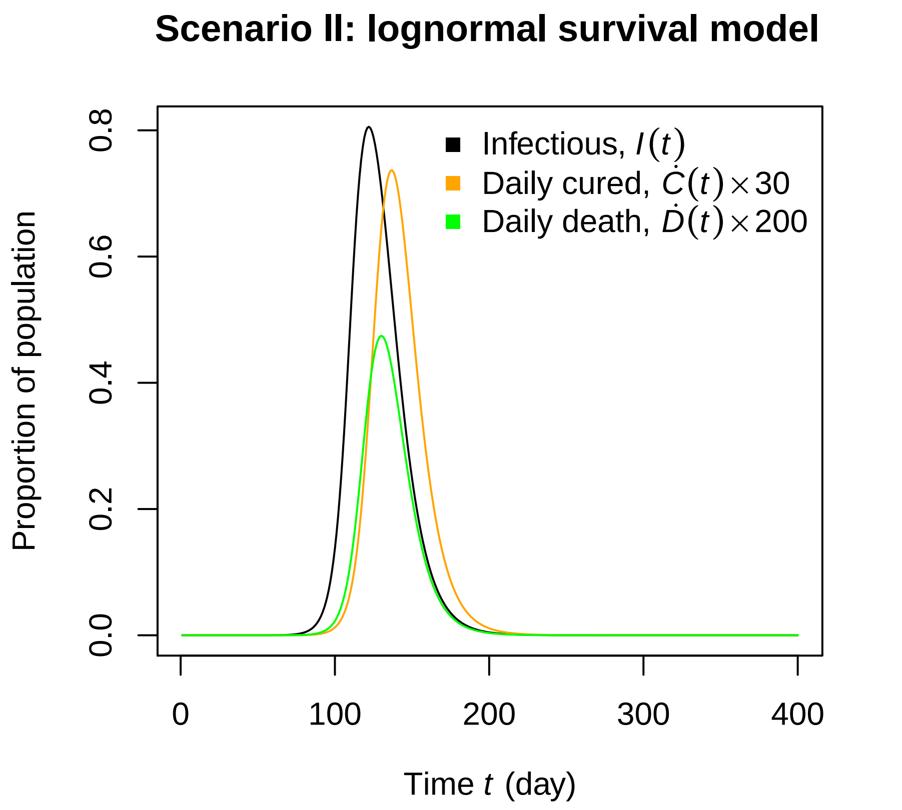

Scenario II: lognormal survival model
Infectious, $I(t)$
Daily cured, $\dot{C}(t) \times 30$
Daily death, $\dot{D}(t) \times 200$
Proportion of population
0.0
0.2
0.4
0.6
0.8
0
100
200
300
400
Time $t$ (day)

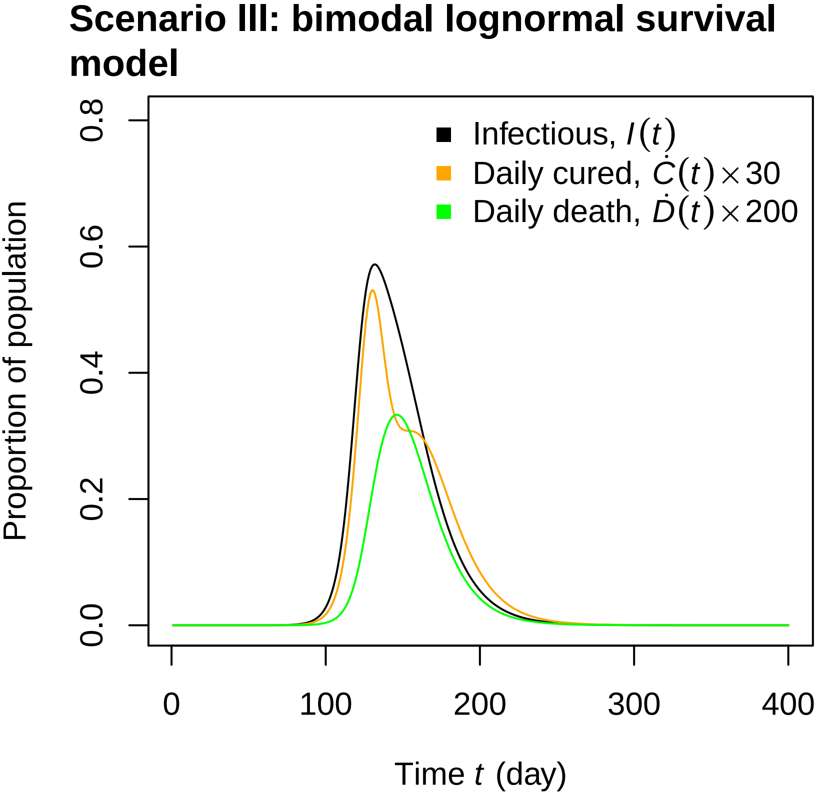
Scenario III: bimodal lognormal survival model
Infectious, $I(t)$
Daily cured, $\dot{C}(t) \times 30$
Daily death, $\dot{D}(t) \times 200$
Proportion of population
0.0
0.2
0.4
0.6
0.8
0
100
200
300
400
Time $t$ (day)
