# Supplementary material for: Cure and death play a role in understanding dynamics for COVID-19: Data-driven competing risk compartmental models, with and without vaccination
Source: PLoS One. 2021 Jul 15;16(7):e0254397. doi: 10.1371/journal.pone.0254397 (PMC8282006; doi:10.1371/journal.pone.0254397)
Supplement: S4 Fig — (A) Daily cured. (B) Daily deaths. (C) aCDR. (D) Infectious cases as percentage of population, I(t)/N. Values of aCDR should be very low at onset of disease due to few cures and death occuring immediately after infection. Therefore, scenario I is unrealistic. (PDF) [file pone.0254397.s007.pdf]

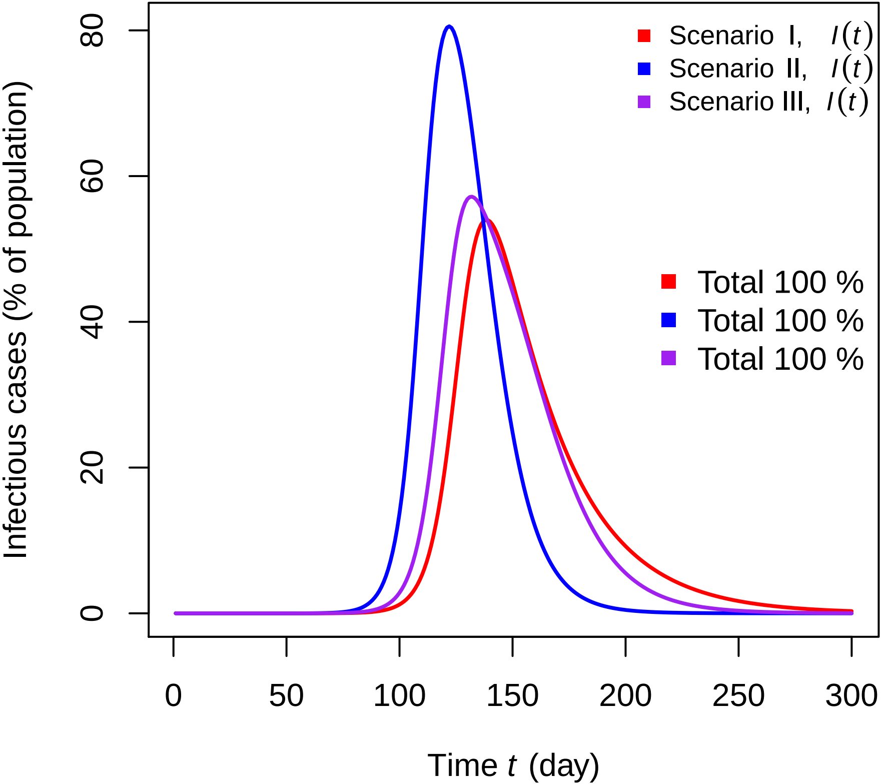

Scenario I, $I(t)$
Scenario II, $I(t)$
Scenario III, $I(t)$
Total 100 %
Total 100 %
Total 100 %
0
20
40
60
80
Infectious cases (% of population)
0
50
100
150
200
250
300
Time t (day)

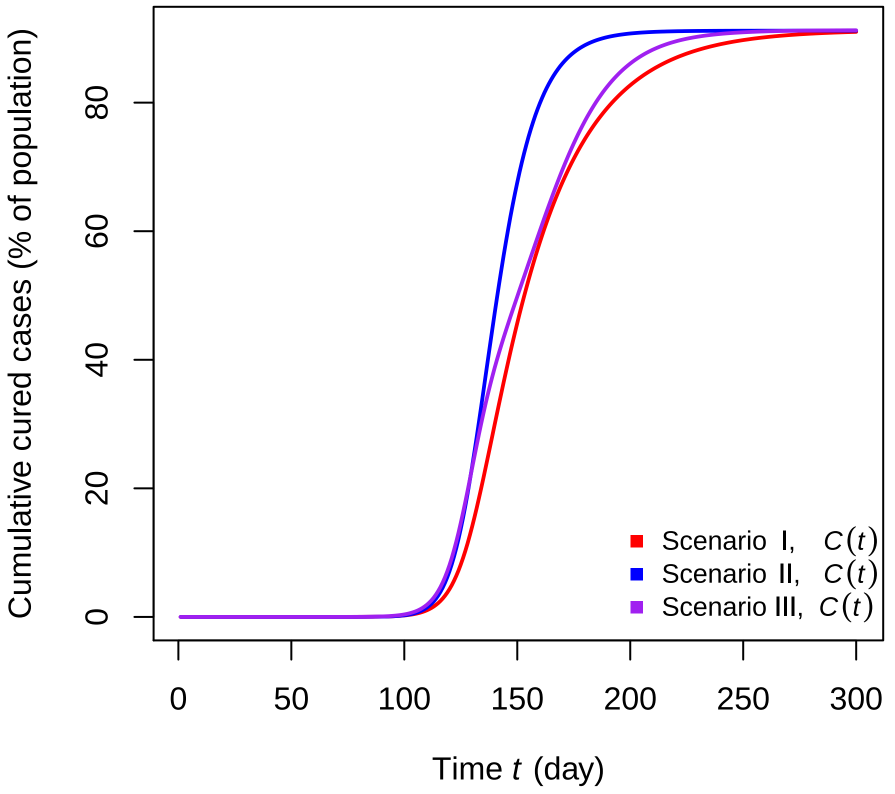

Cumulative cured cases (% of population)
0
20
40
60
80
0
50
100
150
200
250
300
Time t (day)
Scenario I, $C(t)$
Scenario II, $C(t)$
Scenario III, $C(t)$

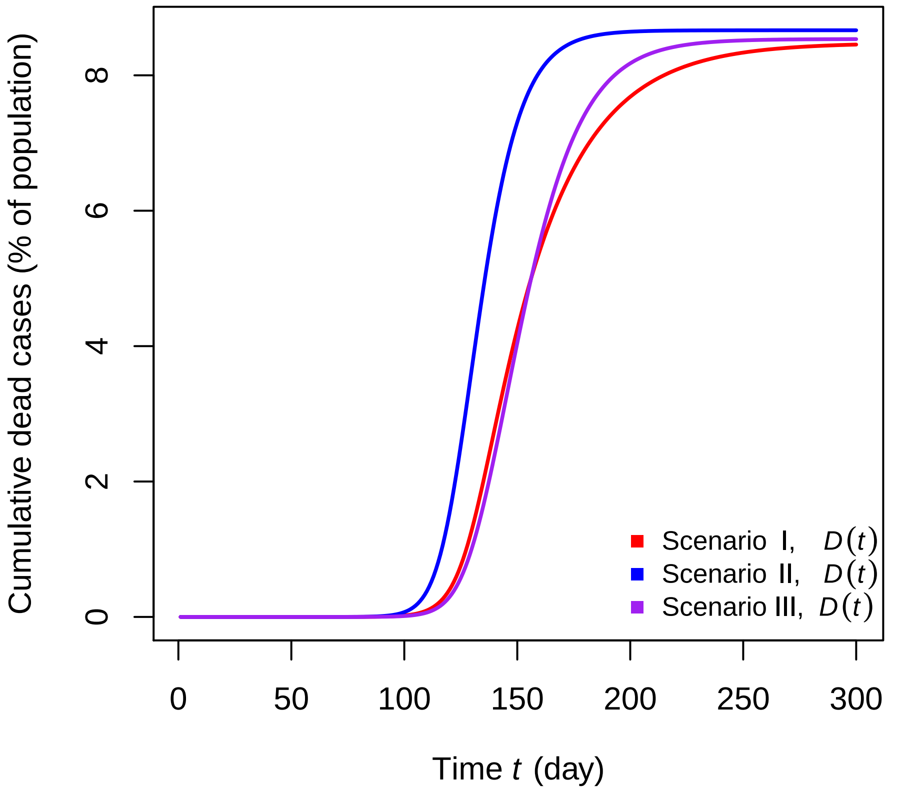

Cumulative dead cases (% of population)
0
2
4
6
8
0
50
100
150
200
250
300
Time t (day)
Scenario I, $D(t)$
Scenario II, $D(t)$
Scenario III, $D(t)$

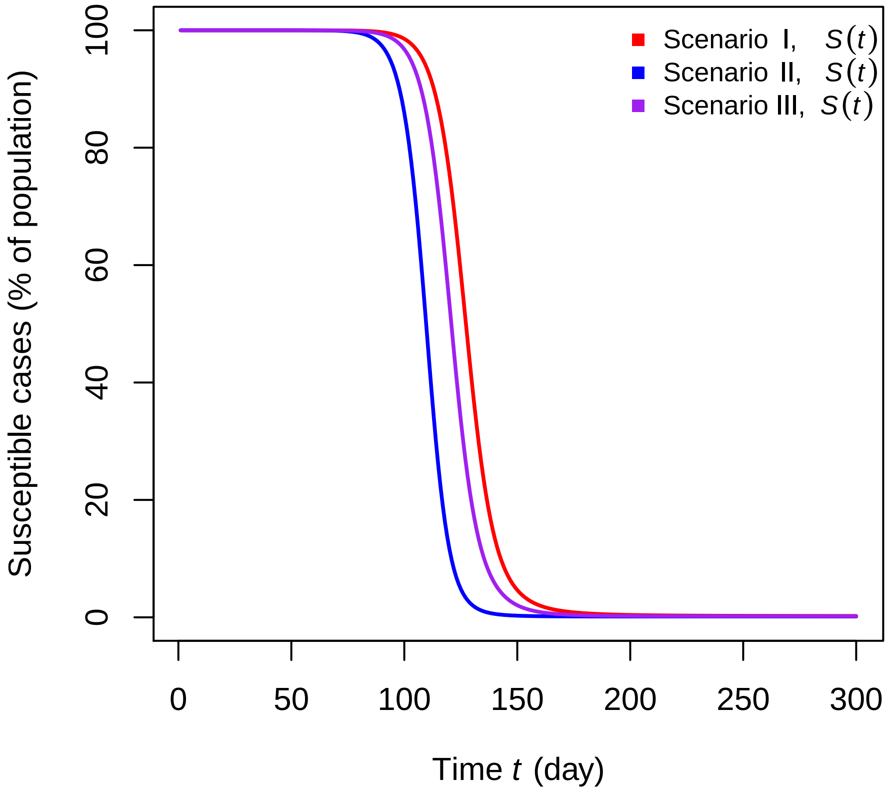

Scenario I, $S(t)$
Scenario II, $S(t)$
Scenario III, $S(t)$
100
80
60
40
20
0
Susceptible cases (% of population)
0
50
100
150
200
250
300
Time t (day)

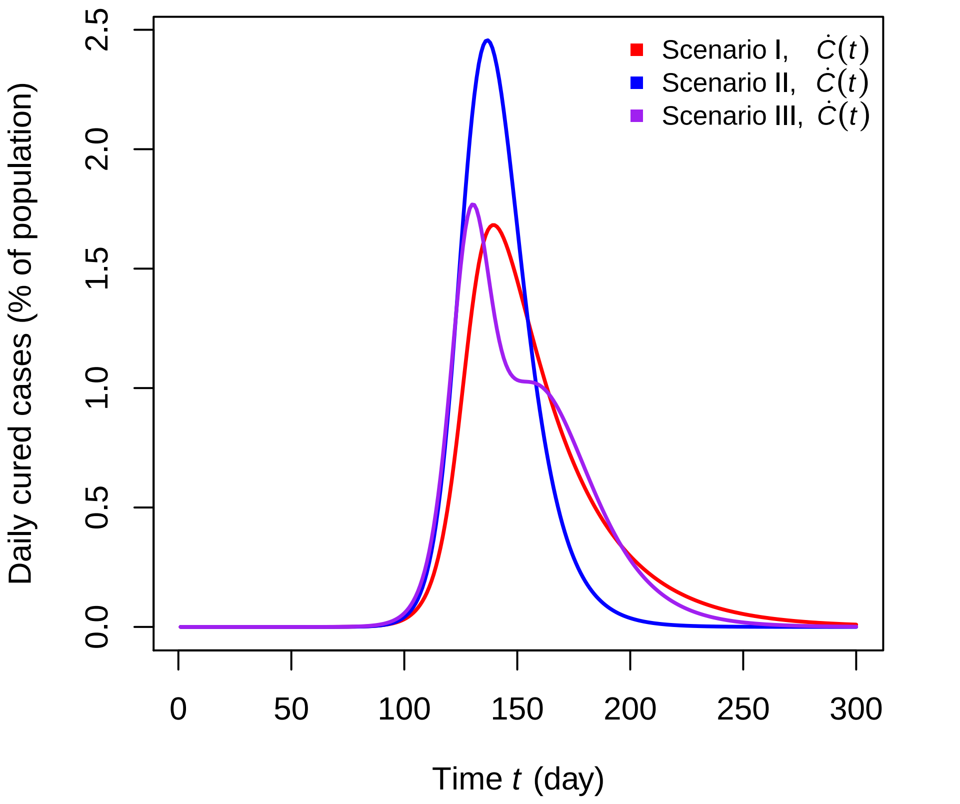
Scenario I, $\dot{C}(t)$
Scenario II, $\dot{C}(t)$
Scenario III, $\dot{C}(t)$
Daily cured cases (% of population)
0.0
0.5
1.0
1.5
2.0
2.5
0
50
100
150
200
250
300
Time t (day)

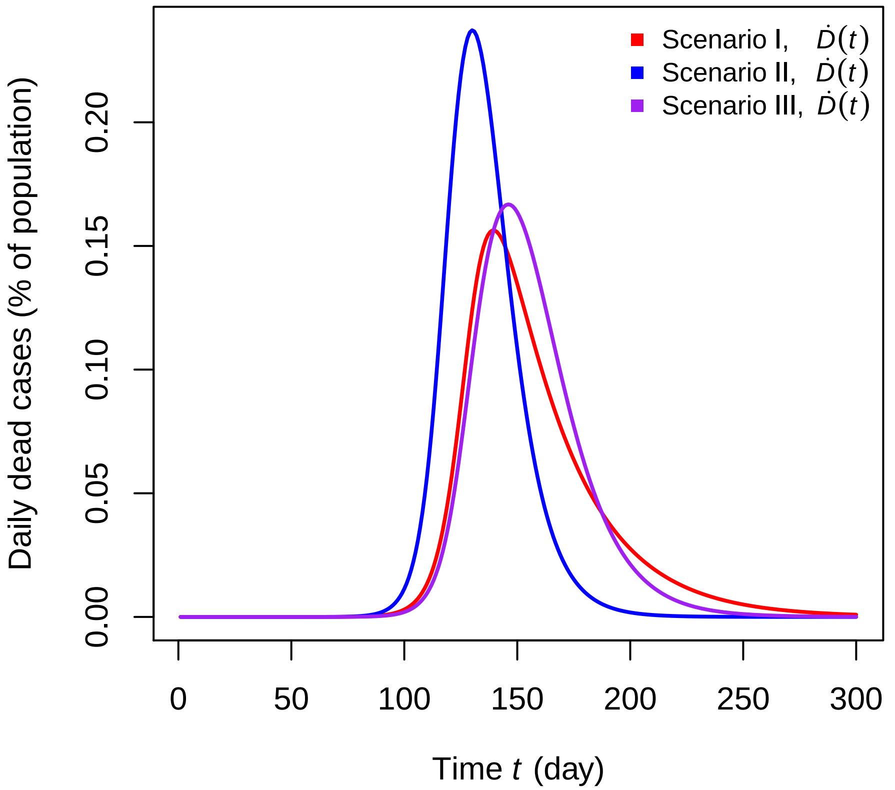

Scenario I, $\dot{D}(t)$
Scenario II, $\dot{D}(t)$
Scenario III, $\dot{D}(t)$
Daily dead cases (% of population)
0.00
0.05
0.10
0.15
0.20
0
50
100
150
200
250
300
Time t (day)

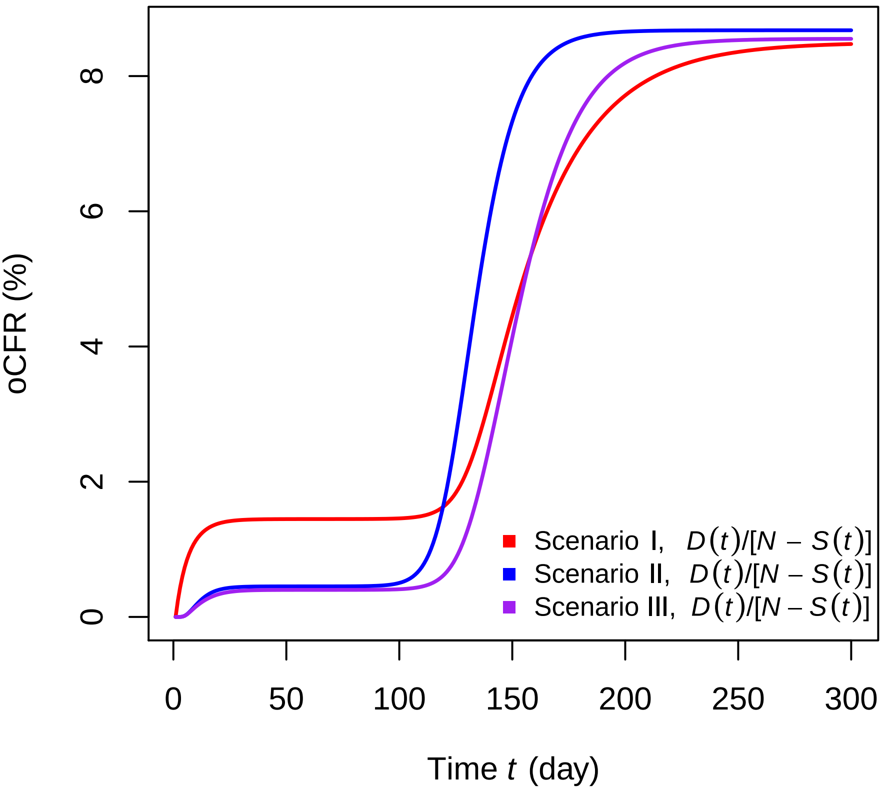

oCFR (%)
0
2
4
6
8
0
50
100
150
200
250
300
Time t (day)
Scenario I, $D(t)/[N-S(t)]$
Scenario II, $D(t)/[N-S(t)]$
Scenario III, $D(t)/[N-S(t)]$
