# Supplementary figures and images for: Cure and death play a role in understanding dynamics for COVID-19: Data-driven competing risk compartmental models, with and without vaccination
Source: PLoS One. 2021 Jul 15;16(7):e0254397. doi: 10.1371/journal.pone.0254397 (PMC8282006; doi:10.1371/journal.pone.0254397)

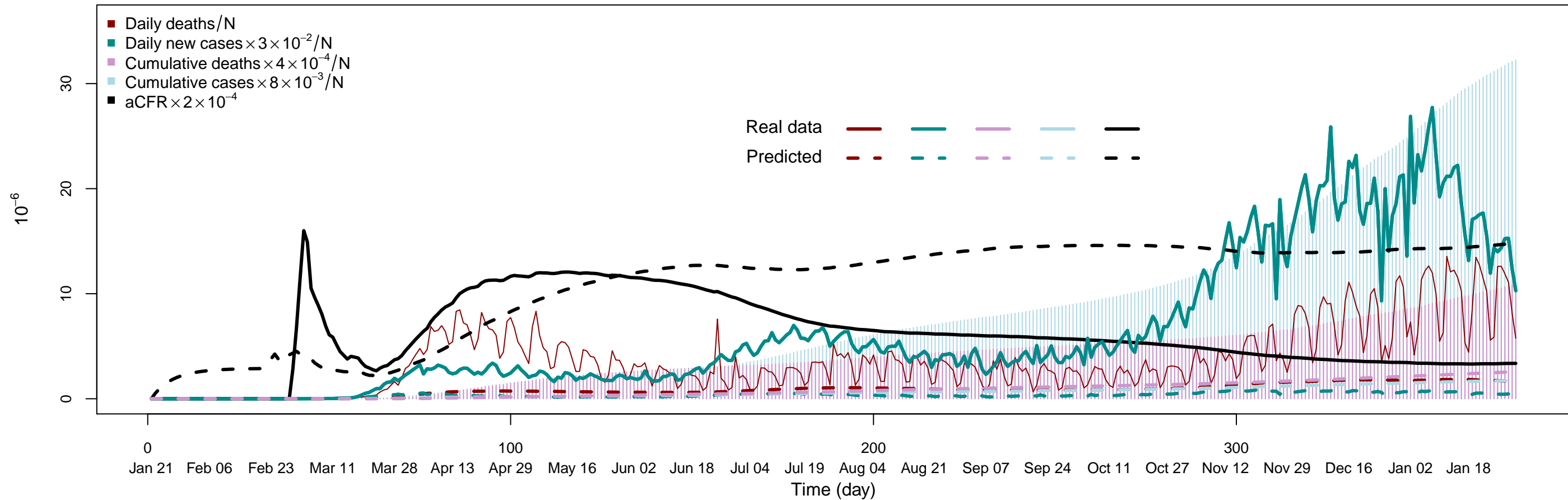

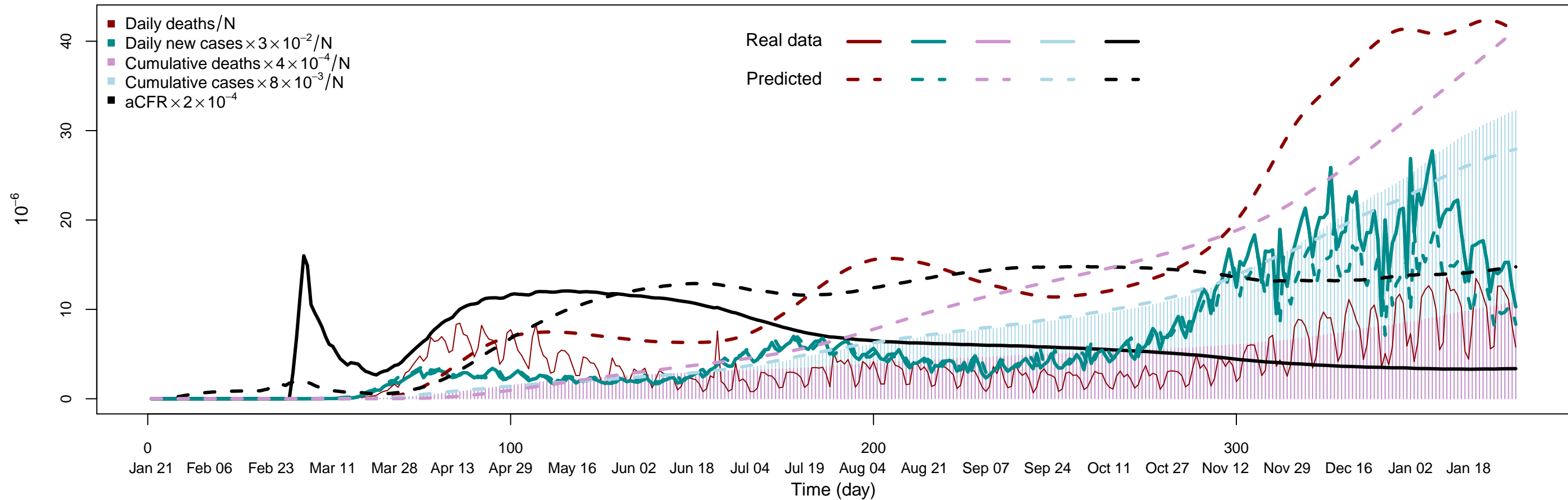

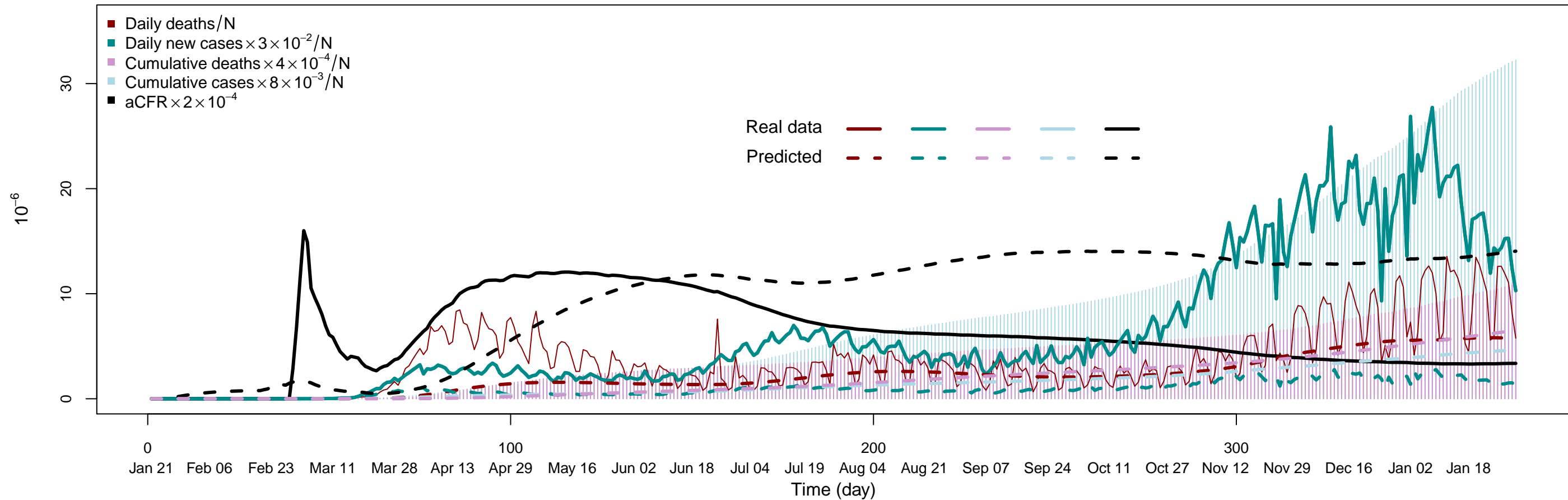

Supplement: S5 Fig — Scenario II is best at estimating daily new cases. However, aCDR and daily deaths are overestimated after first wave, thus suggesting a lower mortality for post-first wave data. (PDf) [file pone.0254397.s008.pdf]

Basic reproductive number  $R_0(t)$

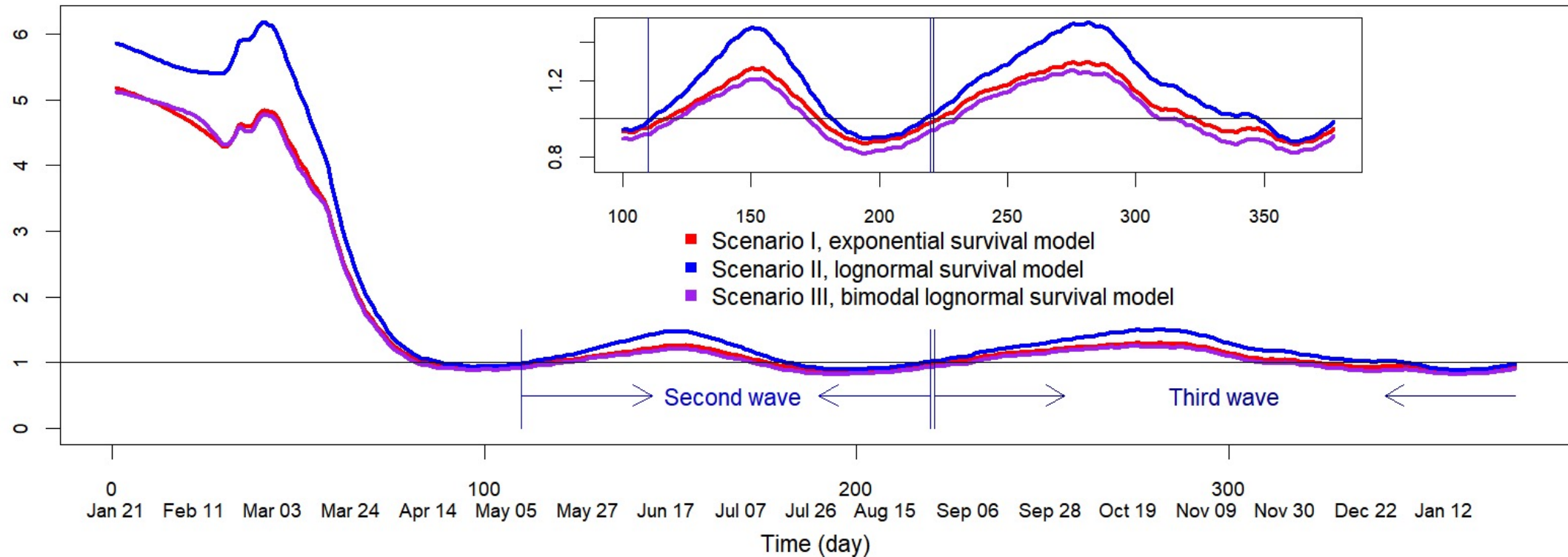

Supplement: S6 Fig — (A) Basic reproduction number R0(t); note its values are much smaller for Scenarios I and III than II. (B) Even though I and III have similar R0(t) profiles, estimated values for daily new infections and deaths are different. (C) Bimodal lognormal distribution continues to perform poorly even under assumption of lower mortality for post-first wave data. (ZIP) [file pone.0254397.s009.zip › S6a_Fig-eps-converted-to.pdf]

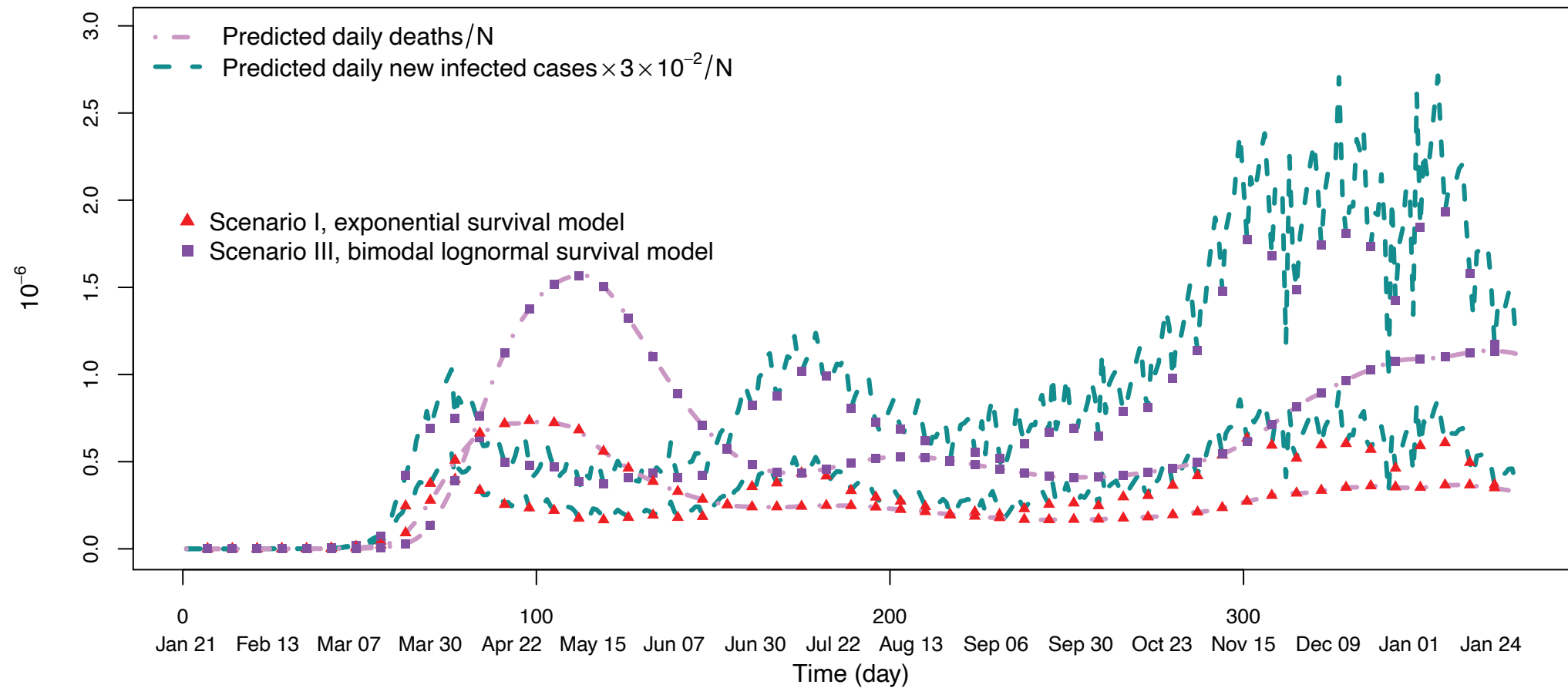

Supplement: S6 Fig — (A) Basic reproduction number R0(t); note its values are much smaller for Scenarios I and III than II. (B) Even though I and III have similar R0(t) profiles, estimated values for daily new infections and deaths are different. (C) Bimodal lognormal distribution continues to perform poorly even under assumption of lower mortality for post-first wave data. (ZIP) [file pone.0254397.s009.zip › S6b_Fig-eps-converted-to.pdf]
