# Supplementary material for: Cure and death play a role in understanding dynamics for COVID-19: Data-driven competing risk compartmental models, with and without vaccination
Source: PLoS One. 2021 Jul 15;16(7):e0254397. doi: 10.1371/journal.pone.0254397 (PMC8282006; doi:10.1371/journal.pone.0254397)
Supplement: S6 Fig — (A) Basic reproduction number R0(t); note its values are much smaller for Scenarios I and III than II. (B) Even though I and III have similar R0(t) profiles, estimated values for daily new infections and deaths are different. (C) Bimodal lognormal distribution continues to perform poorly even under assumption of lower mortality for post-first wave data. (ZIP) [file pone.0254397.s009.zip › S6c_Fig-eps-converted-to.pdf]

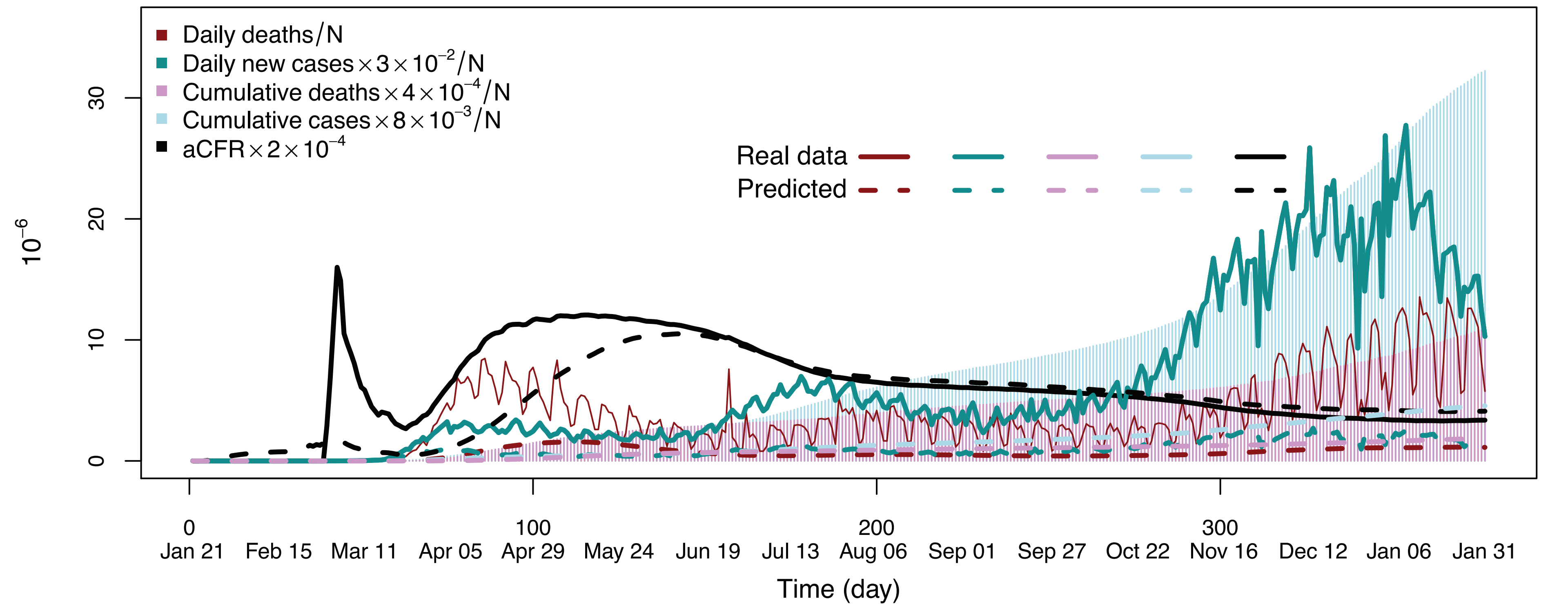

Daily deaths/N
Daily new cases × 3 × 10^−2/N
Cumulative deaths × 4 × 10^−4/N
Cumulative cases × 8 × 10^−3/N
aCFR × 2 × 10^−4
Real data
Predicted
10^−6
0
10
20
30
0
100
200
300
Jan 21
Feb 15
Mar 11
Apr 05
Apr 29
May 24
Jun 19
Jul 13
Aug 06
Sep 01
Sep 27
Oct 22
Nov 16
Dec 12
Jan 06
Jan 31
Time (day)
